# Association of Dexmedetomidine With New-Onset Atrial Fibrillation in Patients With Critical Illness

**DOI:** 10.1001/jamanetworkopen.2023.9955

**Published:** 2023-04-25

**Authors:** Myung Jin Song, Yeonhoon Jang, Ji Hyun Lee, Joo Heung Yoon, Dong Jung Kim, Se Young Jung, Sung Yoon Lim

**Affiliations:** 1 Department of Internal Medicine, Seoul National University Bundang Hospital, Seongnam-si, Republic of Korea; 2 Department of Digital Healthcare, Seoul National University Bundang Hospital, Seongnam-si, Republic of Korea; 3 Cardiovascular Center, Department of Internal Medicine, Seoul National University Bundang Hospital, Seongnam-si, Republic of Korea; 4 Division of Pulmonary, Allergy, and Critical Care Medicine, Department of Medicine, University of Pittsburgh Medical Center, Pittsburgh, Pennsylvania; 5 Department of Cardiovascular and Thoracic Surgery, Seoul National University Bundang Hospital, Seongnam-si, Republic of Korea

## Abstract

**Importance:**

Dexmedetomidine is a widely used sedative in the intensive care unit (ICU) and has unique properties that may be associated with reduced occurrence of new-onset atrial fibrillation (NOAF).

**Objective:**

To investigate whether the use of dexmedetomidine is associated with the incidence of NOAF in patients with critical illness.

**Design, Setting, and Participants:**

This propensity score–matched cohort study was conducted using the Medical Information Mart for Intensive Care-IV database, which includes records of patients admitted to the ICU at Beth Israel Deaconess Medical Center in Boston dating 2008 through 2019. Included patients were those aged 18 years or older and hospitalized in the ICU. Data were analyzed from March through May 2022.

**Exposure:**

Patients were divided into 2 groups according to dexmedetomidine exposure: those who received dexmedetomidine within 48 hours after ICU admission (dexmedetomidine group) and those who never received dexmedetomidine (no dexmedetomidine group).

**Main Outcomes and Measures:**

The primary outcome was the occurrence of NOAF within 7 days of ICU admission, as defined by the nurse-recorded rhythm status. Secondary outcomes were ICU length of stay, hospital length of stay, and in-hospital mortality.

**Results:**

This study included 22 237 patients before matching (mean [SD] age, 65.9 [16.7] years; 12 350 male patients [55.5%]). After 1:3 propensity score matching, the cohort included 8015 patients (mean [SD] age, 61.0 [17.1] years; 5240 males [65.4%]), among whom 2106 and 5909 patients were in the dexmedetomidine and no dexmedetomidine groups, respectively. Use of dexmedetomidine was associated with a decreased risk of NOAF (371 patients [17.6%] vs 1323 patients [22.4%]; hazard ratio, 0.80; 95% CI, 0.71-0.90). Although patients in the dexmedetomidine group had longer median (IQR) length of stays in the ICU (4.0 [2.7-6.9] days vs 3.5 [2.5-5.9] days; *P* < .001) and hospital (10.0 [6.6-16.3] days vs 8.8 [5.9-14.0] days; *P* < .001), dexmedetomidine was associated with decreased risk of in-hospital mortality (132 deaths [6.3%] vs 758 deaths [12.8%]; hazard ratio, 0.43; 95% CI, 0.36-0.52).

**Conclusions and Relevance:**

This study found that dexmedetomidine was associated with decreased risk of NOAF in patients with critical illness, suggesting that it may be necessary and warranted to evaluate this association in future clinical trials.

## Key Points

**Question:**

Is dexmedetomidine use associated with decreased risk of new-onset atrial fibrillation in patients with critical illness?

**Findings:**

In this cohort study including 8015 patients with critical illness, dexmedetomidine was associated with decreased risk of new-onset atrial fibrillation.

**Meaning:**

This study found an association between the use of dexmedetomidine and protection from new-onset atrial fibrillation in patients with critical illness; this finding may warrant further evaluation in a randomized clinical trial.

## Teaser

This cohort study investigates the association of dexmedetomidine use with risk of new-onset atrial fibrillation among patients who are critically ill.

## Introduction

Atrial fibrillation (AF) is the most common type of arrhythmia in patients with critical illness.^1,2^ The reported incidence of new-onset AF (NOAF) varies by study case mix and detection method, ranging from 10% to 44% in patients with sepsis.^3,4^

NOAF in the intensive care unit (ICU) is characterized by rapid structural remodeling due to inflammation that provides an arrhythmogenic atrial substrate and is triggered by potentially reversible factors, such as proarrhythmic drugs, electrolyte imbalance, and volume overload.^5^ The pathogenesis of AF, which involves the autonomic nervous system^6^ and increased sympathetic tone, such as that after surgical procedures or critical illness, is expected to contribute to a high incidence of NOAF among patients in the ICU.^7^ Commonly proposed risk factors associated with NOAF in patients admitted to the ICU include mechanical ventilation, vasoactive drugs, systemic inflammation, and organ dysfunction.^5^ NOAF may cause adverse hemodynamic outcomes, systemic embolism, or stroke, resulting in worse clinical outcomes with longer ICU length of stay (LOS) and higher mortality compared with patients without NOAF.^3,8,9,10^

Dexmedetomidine is a highly selective α2 receptor agonist widely used for sedation in patients with critical illness. It provides sedation by targeting receptors within the locus coeruleus and exerts an analgesic effect via receptors in the spinal cord.^11^ The dose-dependent sedative effect of dexmedetomidine is well understood^12^ and does not result in clinically significant respiratory depression.^13^ Additionally, dexmedetomidine properties that may benefit patients with critical illness include its sympatholytic activity that is associated with reduced heart rate and depressed sinus node and atrial ventricular node conduction.^14^ Furthermore, studies have suggested that dexmedetomidine was associated with cytokine transcription and inhibited inflammation.^15^ These properties collectively suggest that dexmedetomidine may be promising as a treatment associated with decreased risk of NOAF.

Supported by biological plausibility, the preventive outcome associated with dexmedetomidine for NOAF has been largely studied in patients undergoing cardiac surgery, among whom the incidence of NOAF is high.^16^ While results of studies were controversial,^17,18^ a large randomized clinical trial from 2020^19^ failed to show a beneficial effect of dexmedetomidine in reducing the incidence of NOAF. Despite robust evidence developed among patients after cardiac surgery, evidence for an association between dexmedetomidine and NOAF in patients with critical illness beyond those who had undergone cardiac surgery is lacking.

We conducted a retrospective cohort study to investigate the association of dexmedetomidine with NOAF in patients with critical illness. We hypothesized that dexmedetomidine administration may be associated with reduced risk of NOAF in these patients.

## Methods

This cohort study was approved by the Institutional Review Board and Ethics Committee of Seoul National University Bundang Hospital (SNUBH). Because the study was a retrospective records review presenting minimal risk to participants, the institutional review board waived patient informed consent. This study followed the Strengthening the Reporting of Observational Studies in Epidemiology (STROBE) reporting guideline for observational studies.

### Study Design and Population

This study used the Medical Information Mart for Intensive Care (MIMIC)-IV database for primary analysis.^20^ Results were validated by examining those from the ICU cohort of SNUBH (SNUBH-ICU).

**Figure 1.**
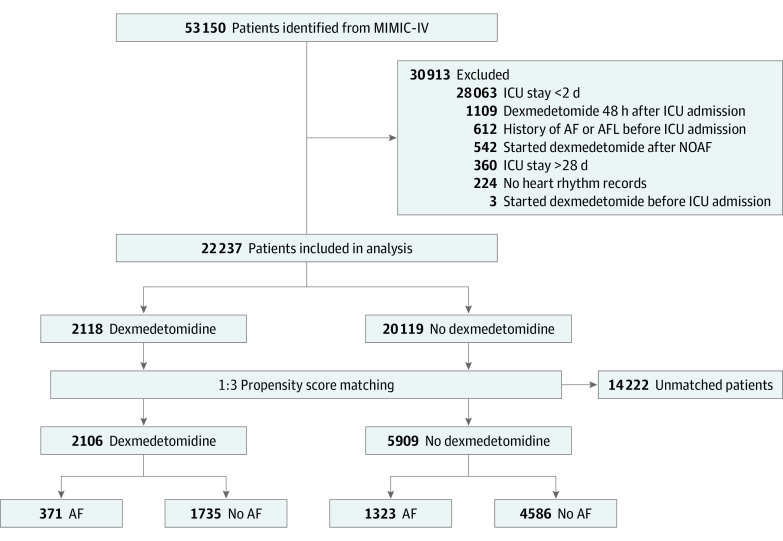
Study Flowchart Patients were identified from the Medical Information Mart for Intensive Care (MIMIC)-IV database. AF indicates atrial fibrillation; AFL, atrial flutter; ICU, intensive care unit; NOAF, NOAF, new-onset atrial fibrillation.

The MIMIC-IV database includes data on 76 540 ICU stays for 53 150 unique patients at Beth Israel Deaconess Medical Center from 2008 through 2019. Patients aged 18 years or older were included. Detailed exclusion criteria are shown in Figure 1. To avoid confounders associated with prolonged ICU stays, the analysis focused on patients who started dexmedetomidine early after ICU admission and excluded patients who started dexmedetomidine 48 hours after ICU admission. Characteristics of patients who started dexmedetomidine 48 hours after ICU admission are summarized in eTable 1 in Supplement 1. Race and ethnicity were assessed because studies have reported an association between race and ethnicity and AF incidence.^21,22^ Race and ethnicity were defined using self-reported demographic data in electronic health records, categorized as African American, American Indian or Alaska Native, Asian, Hispanic, White, other, unable to obtain, and unknown. In this study, except for African American, Asian, Hispanic, and White populations, categories with a small number of patients were combined as other. In the exploratory subgroup analysis, race and ethnicity were divided into 2 groups: African American and not African American (which includes Asian, Hispanic, White, and other). This was done because it has been reported that the prevalence of AF is lower in African American populations compared with other race and ethnicity groups.^21,22^

Eligible patients were divided into 2 groups according to dexmedetomidine exposure: patients who received dexmedetomidine within 48 hours after ICU admission (dexmedetomidine group) and patients who never received dexmedetomidine in the ICU (no dexmedetomidine group). The validation cohort (SNUBH-ICU), consisting of 4568 patients treated between April 1, 2013, and December 31, 2017, was analyzed using the same eligibility criteria as that for the MIMIC-IV database.

### Covariates and Outcomes

Covariates were selected by consensus based on data availability, biological plausibility, and known associations. Detailed information on covariates and missing rates is shown in eMethods 1 and eTables 2 and 3 in Supplement 1. Multivariate imputation by chained equations was used for imputing missing data before propensity score matching.^23^

The primary outcome was the occurrence of NOAF within 7 days of ICU admission. Previously validated nurse-recorded rhythm status from the MIMIC-IV database was used to define NOAF.^24^ Secondary outcomes were ICU LOS, hospital LOS, and in-hospital mortality. Other outcomes of interest were time from ICU admission to NOAF development, adverse events associated with dexmedetomidine (ie, bradycardia and hypotension), and treatment for AF.

### Propensity Score Matching

We used propensity score matching to adjust covariates in modeling the association between use of dexmedetomidine and NOAF. We fitted multivariable logistic regression models to estimate propensity score as the probability of dexmedetomidine use based on prespecified covariates (eTable 2 in Supplement 1). Dexmedetomidine and no dexmedetomidine groups were matched using 1:3 nearest-neighbor matching based on propensity score, with a caliper width of 0.1 SDs or less.^25^ We assessed the covariate balance before and after matching using absolute standardized mean differences (SMDs) and specified an SMD greater than 0.1 as a relevant imbalance.^26^

### Exploratory Subgroup Analyses

We performed subgroup analysis to evaluate the association of dexmedetomidine use with NOAF occurrence in subgroups defined by sex, age (<65 or ≥65 years), race and ethnicity (African American or not African American), ICU unit (cardiovascular ICU or noncardiovascular ICU), sepsis, cardiac surgery, mechanical ventilation support, and continuous kidney replacement therapy. Within each subgroup, we re-estimated the propensity score and performed 1:1 matching.^27^ Heterogeneity was tested across subgroups.

### Sensitivity Analyses

The consistency of results across 3 models was evaluated as follows, adjusting for prespecified covariates: using a multivariable Cox proportional hazard model to estimate hazard ratios (HRs) of study outcomes in the entire cohort, using a multivariable Cox proportional hazard model after imputation of missing data using multivariate imputation by chained equations, and adjusting covariates considering inverse probability of treatment weighting. Because the change in outcome associated with dexmedetomidine may have varied by the duration of dexmedetomidine administration, a series of propensity score matches was performed based on different durations of dexmedetomidine administration (<48 hours, >48 hours and <96 hours, and >96 hours). Owing to the retrospective nature of our study, the data lacked detail about sedation practices, which may have varied with the time of ICU admission. To adjust for differences in sedation practice by time of ICU admission, we performed additional propensity score matching based on the ICU admission period. Thereafter, a sensitivity analysis was conducted to evaluate the incidence of NOAF and in-hospital mortality in different propensity score–matched cohorts.

### Statistical Analysis

Baseline characteristics were presented as mean (SD) or median (IQR) for continuous variables and as No. (percentage) for categorical variables. The χ^2^ test (for categorical variables), Wilcoxon rank sum test (for nonparametric continuous variables), and *t*-test (for parametric continuous variables) were performed to investigate between-group differences.

Differences in time-to-event distributions were evaluated using the log-rank test and visualized using Kaplan-Meier curves. We examined the proportional hazards assumption using Schoenfeld residuals. Results showed no evidence of violation for in-hospital mortality by dexmedetomidine exposure but suggested possible violations for NOAF; therefore, we used flexible parametric survival models for NOAF^28^ and the Cox proportional hazard model for in-hospital mortality to assess associations between dexmedetomidine and each outcome.

Relative excess risk due to interaction (RERI) was calculated to quantify the interaction of no dexmedetomidine and NOAF development in the association with mortality^29,30^ RERI greater than zero indicates a positive interaction and the presence of a synergetic interaction, where the combination of 2 conditions is associated with a larger difference in the outcome than the sum of the individual associations (eMethods 2 in Supplement 1).

Analyses were performed using R statistical software version 4.1.1 (R Project for Statistical Computing). Statistical significance for the 2-sided *P* value was set at <.05. Data were analyzed from March through May 2022.

## Results

### Study Population and Baseline Characteristics

Of 53 150 records, 30 913 records did not meet inclusion eligibility criteria. The remaining eligible MIMIC-IV records for 22 237 patients (mean [SD] age, 65.9 [16.7] years; 12 350 males [55.5%]; 1964 African American [8.8%], 646 Asian [2.9%], 728 Hispanic [3.27%], 14 778 White [66.5%], and 4121 other race or ethnicity [18.5%]) formed the primary cohort, among whom 2118 patients received dexmedetomidine and 20 119 patients did not. The dexmedetomidine group had younger individuals (mean [SD] age, 60.61 [16.02] years vs 66.44 [16.66] years) with a lower mean (SD) Charlson Comorbidity Index score (4.86 [2.83] vs 5.80 [2.95]) but a greater frequency of sepsis and a higher mean sequential organ failure assessment score than those in the no dexmedetomidine group (Table 1). After 1:3 propensity score matching, the matched cohort included 8015 patients (mean [SD] age, 61.0 [17.1] years; 5240 males [65.4%]; 553 African American [6.9%], 163 Asian [2.0%], 237 Hispanic [3.0%], 5,030 White [62.8%], and 2,032 other race or ethnicity [25.4%]), of whom 2106 and 5909 patients were in the dexmedetomidine and no dexmedetomidine groups, respectively (Figure 1). The groups were comparable for all observed variables with SMDs less than 0.1 (eg, mean [SD] age, 60.69 [16.00] years vs 61.12 [17.44] years; SMD = 0.029) (Table 1; eFigure 1 in Supplement 1). In the overall matched cohort, 3131 patients (39.1%) were in the surgical ICU, which accounted for the largest proportion of ICU admissions; 5516 patients (68.8%) were defined as exhibiting sepsis, and 6817 patients (85.1%) required mechanical ventilation support upon ICU admission (Table 1). Sedatives and analgesics other than dexmedetomidine are shown in eTable 4 in Supplement 1.

**Table 1.** Baseline Characteristics of Patients^a^

Characteristic	Before propensity score matching (N = 22 237)			After propensity score matching (N = 8015)		
	Mean (SD)		SMD	Mean (SD)		SMD
	Dexmedetomidine (n = 2118)	No dexmedetomidine (n = 20 119)		Dexmedetomidine (n = 2106)	No dexmedetomidine (n = 5909)	
Age, y	60.61 (16.02)	66.44 (16.66)	0.249	60.69 (16.00)	61.12 (17.44)	0.029
Sex, No. (%)						
Males	1408 (66.48)	10 942 (54.39)	0.357	1398 (66.38)	3842 (65.02)	0.026
Females	710 (33.52)	11 295 (45.61)		708 (33.62)	2067 (34.98)	
Body mass index	29.84 (7.34)	28.88 (7.52)	0.109	29.79 (7.52)	29.72 (7.87)	0.009
Race and ethnicity, No. (%)						
African American	144 (6.80)	1820 (9.05)	0.220	143 (6.79)	410 (6.94)	0.025
Asian	41 (1.94)	605 (3.01)		41 (1.95)	122 (2.06)	
Hispanic	62 (2.93)	666 (3.31)		62 (2.94)	175 (2.96)	
White	1316 (62.13)	13 462 (66.91)		1310 (62.20)	3720 (62.95)	
Other^b^	555 (26.20)	3566 (17.72)		550 (26.12)	1482 (25.08)	
Cardiac surgery, No. (%)	80 (3.78)	859 (4.27)	0.326	80 (3.80)	238 (4.03)	0.012
ICU type, No. (%)						
CCU	110 (5.19)	2593 (12.89)	0.025	110 (5.22)	333 (5.64)	0.048
CVICU	842 (39.75)	3263 (16.22)		834 (39.60)	2217 (37.52)	
MICU	353 (16.67)	3530 (17.55)		351 (16.67)	1031 (17.45)	
SICU	810 (38.24)	9696 (48.19)		808 (38.37)	2323 (39.31)	
Other	3 (0.14)	1037 (5.15)		3 (0.14)	5 (0.08)	
CCI score	4.86 (2.83)	5.80 (2.95)	0.650	4.87 (2.83)	4.96 (2.86)	0.032
Vasoactive-inotropic score^c^	8.34 (23.28)	6.95 (22.13)	0.020	8.29 (23.30)	8.14 (22.84)	0.007
Mechanical ventilation, No. (%)	1983 (93.63)	15 947 (79.26)	0.429	1801 (85.52)	5016 (84.89)	0.018
CKRT, No. (%)	24 (1.13)	249 (1.24)	0.010	72 (3.42)	222 (3.76)	0.018
Sepsis, No. (%)	1467 (69.26)	11 499 (57.15)	0.253	1455 (69.09)	4061 (68.73)	0.008
SOFA score	6.72 (3.51)	5.41 (3.69)	0.366	6.70 (3.50)	6.56 (3.86)	0.038
SAPS II	37.70 (12.99)	37.00 (13.90)	0.052	37.68 (12.96)	37.37 (14.77)	0.022
Initial vital signs at ICU admission						
Mean artery pressure, mm Hg	57.81 (11.54)	58.82 (13.93)	0.079	57.80 (11.55)	57.94 (12.98)	0.011
Pulse rate, beats/min	103.84 (18.49)	104.51 (21.30)	0.033	103.84 (18.47)	104.13 (19.97)	0.015
Respiratory rate, breaths/min	28.22 (6.64)	28.09 (6.45)	0.020	28.19 (6.60)	28.11 (6.58)	0.011
Temperature, °C	37.67 (0.73)	37.43 (0.75)	0.327	37.65 (0.72)	37.62 (0.82)	0.033
Spo_2_ level, %	92.77 (5.22)	91.84 (5.87)	0.168	92.77 (5.24)	92.69 (4.74)	0.016
Laboratory data on day of ICU admission						
pH	7.31 (0.09)	7.32 (0.11)	0.171	7.31 (0.09)	7.31 (0.10)	0.030
po_2_ level, mm Hg	99.02 (57.50)	103.13 (60.06)	0.070	99.89 (59.01)	100.60 (53.46)	0.013
pCo_2_ level, mm Hg	49.08 (11.97)	46.53 (13.83)	0.198	48.60 (11.92)	48.07 (14.02)	0.041
White blood cell count, /μl	16 090 (9020)	14 650 (11 390)	0.140	16 040 (9020)	16 000 (11 650)	0.004
Hemoglobin level, g/dL	9.86 (2.10)	10.23 (2.29)	0.167	9.86 (2.09)	9.92 (2.28)	0.028
Platelet count, ×10^3^/μl	165.83 (85.67)	189.96 (101.98)	0.256	166.22 (85.64)	168.61 (95.19)	0.026
Creatinine level, mg/dL	1.32 (1.24)	1.56 (1.69)	0.159	1.32 (1.25)	1.33 (1.08)	0.010
Blood urea nitrogen level, mg/dL	22.50 (16.61)	28.52 (23.16)	0.299	22.52 (16.62)	22.79 (15.11)	0.017
Lactate level, mg/dL	26.22 (20.54)	24.86 (21.53)	0.063	25.77 (20.27)	25.50 (19.61)	0.011
Sodium level, mEq/L	140.39 (4.33)	139.84 (5.20)	0.116	140.38 (4.33)	140.26 (4.90)	0.027
Chloride level, mEq/L	107.30 (5.60)	105.90 (6.55)	0.230	107.28 (5.60)	107.14 (6.06)	0.022
Potassium level, mEq/L	4.65 (0.79)	4.57 (0.84)	0.101	4.65 (0.79)	4.64 (0.86)	0.007
Calcium level, mg/dL	8.04 (0.83)	8.11 (0.88)	0.082	8.05 (0.82)	8.06 (0.85)	0.017
Bicarbonate level, mEq/L	21.25 (4.19)	21.60 (4.73)	0.078	21.27 (4.18)	21.27 (4.45)	<0.001
Magnesium level, mg/dL	1.94 (0.39)	1.87 (0.35)	0.180	1.95 (0.40)	1.94 (0.39)	0.033

Abbreviations: CCI, Charlson Comorbidity Index; CCU, coronary care unit; CKRT, continuous kidney replacement therapy; CVICU, cardiovascular intensive care unit; ICU, intensive care unit; MICU, medical intensive care unit; MIMIC, Medical Information Mart for Intensive Care; pCo_2_, partial pressure of carbon dioxide; po_2_, partial pressure of oxygen; SAPS, Simplified Acute Physiology Score; SICU, surgical intensive care unit; SMD, standardized mean difference; SOFA, Sequential Organ Failure Assessment; Spo_2_, oxygen saturation as measured by pulse oximetry.

SI conversion factors: To convert bicarbonate, chloride, potassium, and sodium to millimoles per liter, multiply by 1.0; blood urea nitrogen to millimoles per liter, multiply by 0.357; calcium to millimoles per liter, multiply by 0.25; creatinine to micromoles per liter, multiply by 88.4; hemoglobin to grams per liter, multiply by 10.0; lactate to millimoles per liter, multiply by 0.111; magnesium to millimoles per liter, multiply by 0.4114; platelet count to ×10^9^ per liter, multiply by 1.0; white blood cell count to ×10^9^ per liter, multiply by 0.001.

^a^ Patients are from the Medical Information Mart for Intensive Care (MIMIC)-IV database.

^b^ Other indicates race and ethnicity categories queried as American Indian or Alaska Native, unable to obtain, unknown, and other from the MIMIC-IV database.

^c^ The vasoactive-inotropic score was calculated as follows: dopamine dose (in micrograms per kilogram per minute) + dobutamine dose (in micrograms per kilogram per minute) + 100 × epinephrine dose (in micrograms per kilogram per minute) + 10 × milrinone dose (in micrograms per kilogram per minute) + 10 000 × vasopressin dose (in international units per kilogram per minute) + 100 × norepinephrine dose (in micrograms per kilogram per minute).

### Incidence of NOAF and Other Descriptive Outcomes Associated With NOAF

**Figure 2.**
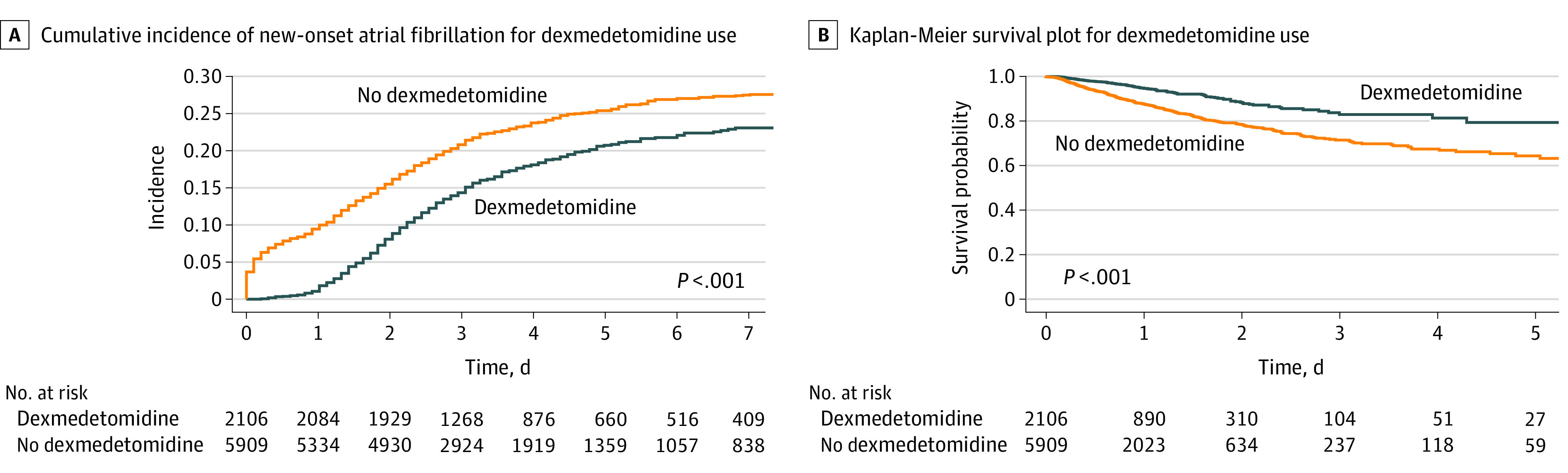
Cumulative Incidence of New-Onset Atrial Fibrillation and Kaplan-Meier Survival Plot for Dexmedetomidine Use Data are from the Medical Information Mart for Intensive Care (MIMIC)-IV database.

The incidence of NOAF was 371 patients (17.6%) in the dexmedetomidine group and 1323 patients (22.4%) in the no dexmedetomidine group. Dexmedetomidine administration was associated with reduced risk of NOAF, with an HR of 0.80 (95% CI, 0.71-0.90) (Table 2). The association remained in sensitivity analyses (eTables 8-12 in Supplement 1). The cumulative incidence plot for NOAF is shown in Figure 2A.

**Table 2.** Outcomes of Interest

Outcome	Patients, No. (%)^a^			*P* value
	Total	Dexmedetomidine	No dexmedetomidine	
Total matched cohort, No.	8015	2106	5909	
NOAF^b^	1694 (21.1)	371 (17.6)	1323 (22.4)	<.001
ICU LOS, median (IQR), d	3.67 (2.59-6.11)	4.00 (2.73-6.90)	3.54 (2.55-5.88)	<.001
Hospital LOS, median (IQR), d	8.99 (6.06-14.81)	9.96 (6.59-16.27)	8.78 (5.89-14.04)	<.001
In-hospital mortality^c^	890 (11.10)	132 (6.27)	758 (12.83)	<.001
Adverse events				
Bradycardia^d^	1252 (15.62)	352 (16.71)	900 (15.23)	.12
Hypotension^e^	658 (8.12)	174 (8.26)	484 (8.19)	.96
With NOAF, No.	1694	371	1323	
Time to development of AF, median (IQR), d	1.49 (0.38-2.36)	2.06 (1.47-2.89)	1.27 (0.17-2.21)	<.001
Treatment for AF				
Rate-control medication^f^	64 (3.78)	19 (5.12)	45 (3.40)	.17
Rhythm-control medication^g^	1071 (63.22)	264 (71.16)	807 (61.00)	.99
Cardioversion	91 (5.37)	19 (5.12)	72 (5.44)	.91

Abbreviations: ICU, intensive care unit; LOS, length of stay; NOAF; new-onset atrial fibrillation.

^a^ Patients are from the Medical Information Mart for Intensive Care (MIMIC)-IV database.

^b^ The hazard ratio for NOAF for dexmedetomidine vs no dexmedetomidine groups, estimated using a flexible parametric survival model, was 0.80 (95% CI, 0.71-0.90).

^c^ The hazard ratio for in-hospital mortality for dexmedetomidine vs no dexmedetomidine groups, estimated using a univariable Cox proportional hazard model, was 0.43 (95% CI, 0.36-0.52).

^d^ Pulse rate less than 50 beats/min.

^e^ Systolic blood pressure less than 90 mm Hg.

^f^ Rate control medication: diltiazem, esmolol, metoprolol, and digoxin.

^g^ Rhythm control medication: amiodarone, magnesium, sotalol, dronedarone, propafenone, and flecainide.

To evaluate the dose-dependent association of dexmedetomidine with NOAF, the cumulative dose of dexmedetomidine from ICU admission to day 7 in the ICU was stratified into 4 groups. A decreasing trend for NOAF incidence with higher cumulative dexmedetomidine doses was observed (eFigure 2 in Supplement 1).

Among patients who developed NOAF, the median (IQR) time from ICU admission to AF onset was significantly longer in the dexmedetomidine group than the no dexmedetomidine group (2.1 [1.5-2.9] days vs 1.3 [0.2-2.2] days; *P* < .001). Regarding treatment for AF, no significant differences were observed in the use of medications for rate control, rhythm control, or electrical cardioversion.

### Associations With LOS and Mortality

Patients in the dexmedetomidine group had a longer median (IQR) LOS in the ICU (4.0 [2.7-6.9] days vs 3.5 [2.5-5.9] days; *P* < .001) and hospital (10.0 [6.6-16.3] days vs 8.8 [5.9-14.0] days; *P* < .001) (Table 2). However, in-hospital mortality was significantly lower in the dexmedetomidine group vs the no dexmedetomidine group (132 patients [6.3%] vs 758 patients [12.8%]; *P* < .001), with an HR of 0.43 (95% CI, 0.36-0.52) (Figure 2B). This association remained in sensitivity analyses (eTables 8, 9, 10, and 12 in Supplement 1). There was an additive interaction between no dexmedetomidine and NOAF development in the association with risk of mortality calculated by RERI (RERI, 0.89; 95% CI, 0.26-1.53) (eTable 5 in Supplement 1).

### Exploratory Subgroup Analyses

**Figure 3.**
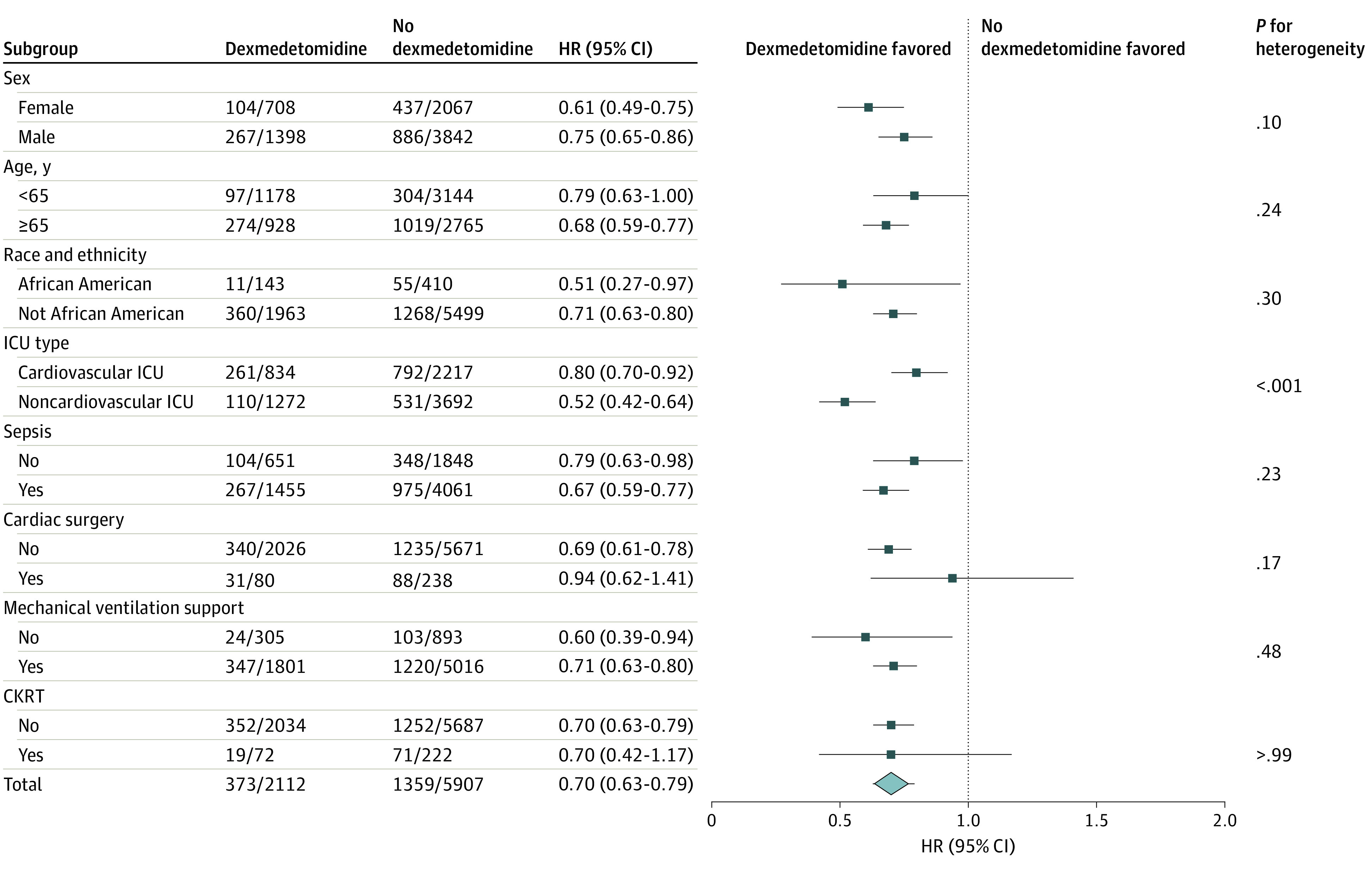
Subgroup Analyses and Interaction Terms for New-Onset Atrial Fibrillation CKRT indicates continuous kidney replacement therapy; HR, hazard ratio; ICU, intensive care unit.

The associations of dexmedetomidine use with NOAF development were consistent across all predefined subgroups but showed significant heterogeneity by type of ICU unit (Figure 3). The risk of NOAF associated with dexmedetomidine was significantly lower in those who were admitted to the noncardiovascular ICU than in those admitted to the cardiovascular ICU (HR, 0.44; 95% CI, 0.35-0.56 vs HR, 0.87; 95% CI, 0.74-1.03; *P* for heterogeneity < .001).

### Validation in SNUBH-ICU Cohort

Validation analysis included 3733 patients admitted to the SNUBH-ICU (mean [SD] age, 64.4 [16.5] years; 12 350 males [58.1%]). After 1:2 propensity score matching, we identified 580 patients in the dexmedetomidine group and 1005 patients in the no dexmedetomidine group. Baseline characteristics were well balanced (SMD<0.1; eg, mean [SD] age, 64.12 [15.66] years vs 64.79 [16.63] years; SMD = 0.041) in the propensity score–matched cohort (eTable 6 and Figures 3-4 in Supplement 1). Compared with the matched primary cohort, the matched validation cohort had a higher mean Simplified Acute Physiology Score (SAPS) II and vasoactive-inotropic score. Unlike in the primary cohort, the medical ICU accounted for the largest proportion of ICU admissions (609 admissions in the overall matched validation cohort [38.4%]) (eTable 6 in Supplement 1).

The incidence of NOAF was 85 patients (11.6%) in the dexmedetomidine group and 277 patients (25.7%) in the no dexmedetomidine group. The dexmedetomidine group had a significantly lower incidence of NOAF than the no dexmedetomidine group, with an HR of 0.64 (95% CI, 0.49-0.83) (eFigure 5 and eTable 7 in Supplement 1). However, in-hospital mortality did not differ significantly between the dexmedetomidine and no dexmedetomidine groups (117 patients [20.2%] vs 232 patients [23.1%]; *P* = .18) (eFigure 5 and eTable 7 in Supplement 1). The association remained in sensitivity analyses (eTable 10 and eTable 12 in Supplement 1).

## Discussion

This cohort study found that administration of dexmedetomidine was associated with reduced risk of NOAF in patients with critical illness. This finding was consistent across 2 tertiary referral hospitals in the US (MIMIC-IV) and South Korea (SNUBH-ICU). Dexmedetomidine was associated with a greater increase in risk of NOAF at the higher dose and with decreased risk of in-hospital mortality in the MIMIC-IV database. However, no association with mortality was observed in the SNUBH-ICU cohort.

The anti-inflammatory and sympatholytic properties of dexmedetomidine suggest that it may be associated with prevention of the development of NOAF. This concept has led several studies to evaluate the association of dexmedetomidine with preventive outcomes for AF in a population with a high incidence of NOAF: patients who had undergone cardiac surgery.^16^ Among those patients, 3 randomized clinical trials were primarily designed to evaluate the effect of dexmedetomidine on AF. Of those studies, 2 reported that dexmedetomidine reduced postoperative NOAF.^31,32^ However, these studies were underpowered owing to the small number of patients (88 and 123 patients, respectively) in their populations. The Dexmedetomidine for Reduction of Atrial Fibrillation and Delirium After Cardiac Surgery (DECADE) trial, the largest of the 3 studies, with a total of 795 patients enrolled, failed to show a preventive effect on NOAF.^19^ These studies evaluated effects of dexmedetomidine in patients after they had undergone cardiac surgery, but our study evaluated the association between dexmedetomidine and NOAF in patients with critical illness beyond those who had undergone cardiac surgery.

Using the MIMIC-IV database, we found that dexmedetomidine was associated with decreased risk of NOAF. However, consistent with results of the DECADE trial,^19^ the association of dexmedetomidine with NOAF in our study exhibited significant heterogeneity in the cardiovascular ICU, where patients with cardiac and vascular surgical issues were admitted. This suggests that the potentially beneficial anti-inflammatory and sympatholytic properties of dexmedetomidine may not have been sufficiently effective for patients among whom physical alteration of the cardiac structure resulting from the surgical procedure itself played a major role in the pathophysiology of NOAF.^33^

ICU and hospital LOS were longer among patients treated with dexmedetomidine in our study. Dexmedetomidine, along with propofol and midazolam, is a widely used sedative in the ICU and is known to have analgesic and preventive effects on delirium. As a result, physicians select dexmedetomidine as a primary sedative in patients expected to have weaning difficulty and high risk of delirium.^34^ This may have been associated with the prolonged ICU and hospital stays in the dexmedetomidine group despite propensity score matching of detailed baseline characteristics. Nevertheless, dexmedetomidine administration was associated with decreased risk of not only NOAF, but also mortality in the primary cohort.

A positive additive interaction between no dexmedetomidine and NOAF development in the association with in-hospital mortality was found. This suggests that when patients developed NOAF without administration of dexmedetomidine, there was an additional risk of mortality beyond the combined risk of no dexmedetomidine and NOAF development. Although we cannot clarify the underlying biological mechanisms based on this study’s results, a possible explanation may be that the pleiotropic effect of dexmedetomidine on immunomodulation, sympatholytic activity, and hemodynamics is synergistic in the presence of NOAF.^35^

In contrast, there was no association between dexmedetomidine and in-hospital mortality in the SNUBH-ICU cohort.^36^ This could be explained by differences in the baseline risk of death between cohorts. The SAPS II score, which represents severity of illness in patients, was higher in the SNUBH-ICU than the MIMIC- IV cohort. Even if dexmedetomidine was associated with a preventive outcome for NOAF, if the overall risk of death from the patient’s main diagnosis was extreme, decreased risk of NOAF with dexmedetomidine administration may still not have been associated with improved mortality. This explanation is corroborated by the post hoc bayesian analysis of the Sedation Practice in Intensive Care Evaluation III trial,^37^ which found that the probability of benefits obtained from dexmedetomidine administration (vs no dexmedetomidine administration) declined as the Acute Physiology and Chronic Health Evaluation II score increased.

### Limitations

This study has several limitations. First, despite propensity score matching to balance observed baseline characteristics between groups, there may be unmeasured confounders influencing the result; these may include medications administered before ICU admission, concomitant medication during ICU care, and cardiac function. Second, the no dexmedetomidine group was defined as patients who never received dexmedetomidine, so sedatives used in this group were heterogenous. This may have influenced the results. Third, dexmedetomidine administration practices were not uniform. To minimize effect modification due to different dexmedetomidine administration practices across patients, we included only patients who started dexmedetomidine within the first 48 hours after ICU admission. Additionally, we performed sensitivity analysis regarding duration of dexmedetomidine administration and time of ICU admission and demonstrated consistency in the results. Fourth, only patients who stayed in the ICU for more than 48 hours were considered; this was done to select patients who were more severely ill and those who were exposed to dexmedetomidine for a sufficient period. Therefore, the findings cannot be translated to patients who left the ICU due to improvements in their health or due to death in the first 48 hours after ICU admission.

## Conclusion

This cohort study found that dexmedetomidine was associated with decreased risk of NOAF in 2 different propensity score–matched ICU cohorts, supporting generalizability of the results. A dose-response characteristic was found in the association between dexmedetomidine and NOAF occurrence, supporting the hypothesized association between dexmedetomidine and NOAF. These findings suggest that use of dexmedetomidine may be associated with a protective outcome against NOAF in patients with critical illness. Further research is needed to evaluate these findings in randomized clinical trials.

## Notes

Supplement 1. **eMethods 1.** Detailed Information on Covariates Used in Propensity Score Matching **eMethods 2.** Formulas and Interpretation of Relative Excess Risk in Interaction **eTable 1.** Baseline Characteristics and Outcomes of Patients Who Started Dexmedetomidine Administration 48 h After Intensive Care Unit Admission in Primary Cohort **eTable 2.** Percentage of Missing Data From Primary Cohort **eTable 3.** Percentage of Missing Data From Validation Cohort **eTable 4.** Sedatives and Analgesics Other Than Dexmedetomidine in Primary Cohort **eTable 5.** Relative Excess Risk in Interaction Between New-Onset Atrial Fibrillation and Dexmedetomidine in Association With In-Hospital Mortality in Primary Cohort **eTable 6.** Baseline Characteristics of Validation Cohort Before and After Propensity Score Matching **eTable 7.** Outcomes of Interest in Validation Cohort **eTable 8.** Cox Proportional Hazard Model in Entire Cohort From Primary Database Before Imputation for Missing Data **eTable 9.** Cox Proportional Hazard Model in Entire Cohort From Primary Database After Imputation for Missing Data **eTable 10.** Association Between Dexmedetomidine Infusion and Outcomes in Different Models **eTable 11.** Sensitivity Analyses for Patients by Duration of Dexmedetomidine Administration in Primary Cohort **eTable 12.** Sensitivity Analyses for Patients by Calendar Period of Intensive Care Unit Admission **eFigure 1.** Absolute Standardized Mean Differences of Individual Covariates Before and After Propensity Score Matching in Primary Cohort **eFigure 2.** Dose-Response Association Between Quartiles of Dexmedetomidine Dose and Incidence of New-Onset Atrial Fibrillation **eFigure 3.** Study Flowchart for Validation Cohort **eFigure 4.** Absolute Standardized Mean Differences of Individual Covariates Before and After Propensity Score Matching in Validation Cohort **eFigure 5.** Cumulative Incidence of New-Onset Atrial Fibrillation and Kaplan-Meier Survival Plot for Dexmedetomidine Use in Validation Cohort
eReferences

Supplement 2.
Data Sharing Statement
