# Supplementary material for: Association of Dexmedetomidine With New-Onset Atrial Fibrillation in Patients With Critical Illness
Source: JAMA Netw Open. 2023 Apr 25;6(4):e239955. doi: 10.1001/jamanetworkopen.2023.9955 (PMC10130948; doi:10.1001/jamanetworkopen.2023.9955)
Supplement: Supplement 1. — eMethods 1. Detailed Information on Covariates Used in Propensity Score Matching eMethods 2. Formulas and Interpretation of Relative Excess Risk in Interaction eTable 1. Baseline Characteristics and Outcomes of Patients Who Started Dexmedetomidine Administration 48 h After Intensive Care Unit Admission in Primary Cohort eTable 2. Percentage of Missing Data From Primary Cohort eTable 3. Percentage of Missing Data From Validation Cohort eTable 4. Sedatives and Analgesics Other Than Dexmedetomidine in Primary Cohort eTable 5. Relative Excess Risk in Interaction Between New-Onset Atrial Fibrillation and Dexmedetomidine in Association With In-Hospital Mortality in Primary Cohort eTable 6. Baseline Characteristics of Validation Cohort Before and After Propensity Score Matching eTable 7. Outcomes of Interest in Validation Cohort eTable 8. Cox Proportional Hazard Model in Entire Cohort From Primary Database Before Imputation for Missing Data eTable 9. Cox Proportional Hazard Model in Entire Cohort From Primary Database After Imputation for Missing Data eTable 10. Association Between Dexmedetomidine Infusion and Outcomes in Different Models eTable 11. Sensitivity Analyses for Patients by Duration of Dexmedetomidine Administration in Primary Cohort eTable 12. Sensitivity Analyses for Patients by Calendar Period of Intensive Care Unit Admission eFigure 1. Absolute Standardized Mean Differences of Individual Covariates Before and After Propensity Score Matching in Primary Cohort eFigure 2. Dose-Response Association Between Quartiles of Dexmedetomidine Dose and Incidence of New-Onset Atrial Fibrillation eFigure 3. Study Flowchart for Validation Cohort eFigure 4. Absolute Standardized Mean Differences of Individual Covariates Before and After Propensity Score Matching in Validation Cohort eFigure 5. Cumulative Incidence of New-Onset Atrial Fibrillation and Kaplan-Meier Survival Plot for Dexmedetomidine Use in Validation Cohort eReferences [file jamanetwopen-e239955-s001.pdf]

Supplemental Online Content

Song MJ, Jang Y, Lee JH, et al. Association of dexmedetomidine with new-onset atrial fibrillation in patients with critical illness. *JAMA Netw Open*. 2023;6(4):e239955. doi:10.1001/jamanetworkopen.2023.9955

eMethods 1. Detailed Information on Covariates Used in Propensity Score Matching

eMethods 2. Formulas and Interpretation of Relative Excess Risk in Interaction

eTable 1. Baseline Characteristics and Outcomes of Patients Who Started Dexmedetomidine Administration 48 h After Intensive Care Unit Admission in Primary Cohort

eTable 2. Percentage of Missing Data From Primary Cohort

eTable 3. Percentage of Missing Data From Validation Cohort

eTable 4. Sedatives and Analgesics Other Than Dexmedetomidine in Primary Cohort

eTable 5. Relative Excess Risk in Interaction Between New-Onset Atrial Fibrillation and Dexmedetomidine in Association With In-Hospital Mortality in Primary Cohort

eTable 6. Baseline Characteristics of Validation Cohort Before and After Propensity Score Matching

eTable 7. Outcomes of Interest in Validation Cohort

eTable 8. Cox Proportional Hazard Model in Entire Cohort From Primary Database Before Imputation for Missing Data

eTable 9. Cox Proportional Hazard Model in Entire Cohort From Primary Database After Imputation for Missing Data

eTable 10. Association Between Dexmedetomidine Infusion and Outcomes in Different Models

eTable 11. Sensitivity Analyses for Patients by Duration of Dexmedetomidine Administration in Primary Cohort

eTable 12. Sensitivity Analyses for Patients by Calendar Period of Intensive Care Unit Admission

eFigure 1. Absolute Standardized Mean Differences of Individual Covariates Before and After Propensity Score Matching in Primary Cohort

eFigure 2. Dose-Response Association Between Quartiles of Dexmedetomidine Dose and Incidence of New-Onset Atrial Fibrillation

eFigure 3. Study Flowchart for Validation Cohort

eFigure 4. Absolute Standardized Mean Differences of Individual Covariates Before and After Propensity Score Matching in Validation Cohort

eFigure 5. Cumulative Incidence of New-Onset Atrial Fibrillation and Kaplan-Meier Survival Plot for Dexmedetomidine Use in Validation Cohort

eReferences

This supplemental material has been provided by the authors to give readers additional information about their work.

© 2023 Song MJ et al. *JAMA Network Open.*

eMethods 1. Detailed Information on Covariates Used in Propensity Score Matching

Covariates were selected by consensus based on data availability, biological plausibility, and known associations. Baseline characteristics recorded within 24 hours after ICU admission were collected, including age, sex, body mass index, race and ethnicity, ICU type, sequential organ failure assessment (SOFA) score, Charlson comorbidity index, and Simplified Acute Physiology Score (SAPS) II. Information regarding MV, continuous kidney replacement therapy (CKRT), administration of vasoactive drugs, and surgery at ICU admission were also recorded. Vasoactive drug doses were converted to a vasoactive-inotropic score (VIS).[1] Sepsis was defined using the Sepsis-3 criteria.[2] The initial vital sign records at ICU admission and laboratory variables measured on the day of ICU admission were extracted.

© 2023 Song MJ et al. *JAMA Network Open.*

eMethods 2. Formulas and Interpretation of Relative Excess Risk in Interaction

Interaction is the situation in which the association of one risk factor with a certain outcome variable differs across strata of another risk factor.[3] The presence and direction of interaction depends on the scale, e.g. additive or multiplicative, that is used. From a public health perspective, the assessment of interaction on an additive scale is the most relevant.

Rothman presents three measures of biological interaction on the additive scale: (i) the relative excess risk due to interaction (RERI), (ii) the attributable proportion due to interaction (AP), and (iii) the synergy index (S)[3,4] In this study we adopted RERI to evaluate the interaction between "no use of dexmedetomidine" and "NOAF development".

Formula

For two dichotomous risk factors ("no use of dexmedetomidine" and "NOAF development"), four categories can be constructed: 1) the reference category (background risk; no risk factors exist), 2) a category for exposure to one of the risk factors (R10), 3) a category for exposure to the other factor (R01), 4) a category for joint exposure to both risk factors (R11).

Subsequently, the measures to evaluate additive interaction in relative risk data can be calculated as follows ('RR', relative risk, refers to either odds ratio in a logistic regression model or hazard ratio in a Cox regression model):

RERI = RR11 – RR10 – RR01 + 1.

In the case of this study, dexmedetomidine is a preventive exposure for mortality. To use the lowest risk group as a reference, we coded "no use of dexmedetomidine" as 1 and "use of dexmedetomidine" as 0. In this way, we could calculate the RERI for the two risk factors. [5]

Interpretation

RERI can go from - infinity to + infinity. RERI = 0 means no interaction or exactly additivity; RERI > 0 means positive interaction or more than additivity; RERI < 0 means negative interaction or less than additivity.

We calculated the measure using the epiR package for R software.

© 2023 Song MJ et al. *JAMA Network Open.*

eTable 1. Baseline Characteristics and Outcomes of Patients Who Started Dexmedetomidine Administration 48 h After Intensive Care Unit Admission in Primary Cohort

Variables	Mean (SD)
	**n = 1,109**
Age, y	57.33 ± 16.88
Sex, No. (%)	
Males	674 (60.78)
Females	435 (39.22)
Body mass index	30.26 ± 8.50
Race and ethnicity, No. (%)	
African American	83 (7.48)
Asian	20 (1.80)
Hispanic	52 (4.69)
White	626 (56.45)
Other	328 (29.58)
Caridac surgery, No. (%)	38 (3.43)
ICU Unit, No. (%)	
CCU	93 (8.39)
CVICU	102 (9.20)
MICU	15 (1.35)
SICU	265 (23.90)
Other	634 (57.17)
Charlson Comorbidity Index	4.73 (3.01)
Mechanical ventilation, No. (%)	1,048 (94.50)
CKRT, No. (%)	39 (3.52)
Sepsis, No. (%)	1,021 (92.06)
SOFA score	8.37 (4.23)
SAPS II	39.61 (14.49)
Initial vital signs at ICU admission	
Mean arterial pressure, mmHg	57.79 (14.42)
Pulse rate, beats/min	110.25 (20.51)
Respiratory rate, breaths/min	29.56 (7.50)
Temperature, °C	37.82 (0.89)
Spo_2 level, %	91.20 (7.31)
Laboratory data on day of ICU admission	
pH	7.29 (0.12)
po_2, mmHg	91.71 (55.62)
pCo_2, mmHg	50.46 (16.40)
White blood cell count, /µl	16,350 ± 10,960
Hemoglobin level, g/dL	10.13 ± 2.39
Platelet count, $\times 10^3$/µl	178.72 ± 104.69
Creatinine level, mg/dL	1.65 ± 1.58
Blood urea nitrogen level, mg/dL	28.09 ± 22.09
Lactate level, mg/dL	29.01 ± 25.05
Sodium level, mEq/L	140.44 ± 5.27
Chloride level, mEq/L	106.08 ± 6.78
Potassium level, mEq/L	4.69 ± 0.90
Calcium level, mg/dL	7.82 ± 0.88
Bicarbonate level, mEq/L	20.49 ± 5.07

© 2023 Song MJ et al. *JAMA Network Open.*

Variables	Mean (SD)
Magnesium level, mg/dL	1.81 ± 0.34
ICU length of stay, median (IQR), d	9.83 (6.67-14.54)
Hospital length of stay, median (IQR), d	17.27 (11.74-25.21)
In-hospital mortality, No.(%)	171 (15.42)

CCU, Coronary care unit; CKRT, Continuous kidney replacement therapy; CVICU, Cardiovascular intensive care unit; ICU, intensive care unit; MICU, Medical intensive care unit; MIMIC, Medical Information Mart for Intensive Care; pCo2, partial pressure of carbon dioxide; po2, partial pressure of oxygen; SAPS, Simplified Acute Physiology Score; SICU, Surgical intensive care unit; SOFA, Sequential Organ Failure Assessment; Spo_2, oxygen saturation as measured by pulse oximetry.

SI conversion factors: To convert bicarbonate, chloride, potassium, and sodium to millimoles per liter, multiply by 1.0; blood urea nitrogen to millimoles per liter, multiply by 0.357; calcium to millimoles per liter, multiply by 0.25; creatinine to micromoles per liter, multiply by 88.4; hemoglobin to grams per liter, multiply by 10.0; lactate to millimoles per liter, multiply by 0.111; magnesium to millimoles per liter, multiply by 0.4114; platelet count to $\times 10^9$ per liter, multiply by 1.0; white blood cell count to $\times 10^9$ per liter, multiply by 0.001.

© 2023 Song MJ et al. *JAMA Network Open.*

eTable 2. Percentage of Missing Data From Primary Cohort

Variables		n=22,237
Age		0%
Sex		0%
Body mass index		40.62%
Race and ethnicity		0%
Charlson Comorbidity Index		0%
Cardiac surgery		0%
ICU Unit		0%
Vasoactive Inotropic Score		0%
Mechanical ventilation		0%
Continuous kidney replacement therapy		0%
Sepsis		0%
SOFA score		0%
SAPS II		0%
Initial vital signs at ICU admission		
	Mean artery pressure	0.23%
	Pulse rate	0.21%
	Respiratory rate	0.34%
	Temperature	4.38%
	Spo_2	0.21%
Laboratory data on day of ICU admission		
	pH	30.95%
	po_2	31.25%
	pCo_2	31.25%
	White blood cell	0.43%
	Hemoglobin	0.46%
	Platelet	0.44%
	Creatinine	0.37%
	Blood urea nitrogen	0.40%
	Lactate	27.09%
	Sodium	0.45%
	Chloride	0.44%
	Potassium	0.53%
	Calcium	7.68%
	Bicarbonate	0.44%
	Magnesium	4.95%

ICU, intensive care unit; MIMIC, Medical Information Mart for Intensive Care; pCo_2, partial pressure of carbon dioxide; po_2, partial pressure of oxygen; SAPS, Simplified Acute Physiology Score; SOFA, Sequential Organ Failure Assessment; Spo_2, oxygen saturation as measured by pulse oximetry.

© 2023 Song MJ et al. *JAMA Network Open.*

eTable 3. Percentage of Missing Data From Validation Cohort

**Variables**		**n=3,733**
Age		0%
Sex		0%
Body mass index		0.21%
ICU unit		0%
Charlson comorbidity index		0%
APACHE II		3%
SAPS II		4%
Vasoactive inotropic score		0%
Mechanical ventilation		0%
Continuous kidney replacement therapy		0%
Cardiac surgery		0%
Initial vital sign at ICU admission		0%
	Mean arterial pressure	0%
	Pulse rate	0%
	Respiratory rate	0.01%
	Temperature	0.01%
	Spo_2	0.01%

APACHE, acute physiology and chronic health evaluation; ICU, intensive care unit; SAPS, Simplified Acute Physiology Score; Spo2, oxygen saturation as measured by pulse oximetry.

© 2023 Song MJ et al. *JAMA Network Open.*

eTable 4. Sedatives and Analgesics Other Than Dexmedetomidine in Primary Cohort

Variables	No. (%)	
	**Dexmedetomidine (n=2,106)**	**No dexmedetomidine (n=5,909)**
Propofol		
Number of patients	1,826 (86.7%)	3,864 (65.4%)
Dose, mean (SD), mg	5162.9 ± 7711.0	3208.8 ± 6694.0
Midazolam		
Number of patients	537 (25.5%)	1,308 (22.1%)
Dose, mean (SD), mg	22.4 ± 136.4	35.5 ± 230.7
Fentanyl		
Number of patients	1467 (69.7%)	2997 (50.7%)
Dose, mean (SD), mg	0.35 ± 0.64	0.30 ± 1.29
Morphine		
Number of patients	574 (27.3%)	1,700 (28.8%)
Dose, mean (SD), mg	4.6 ±12.5	6.1 ± 32.0

© 2023 Song MJ et al. *JAMA Network Open.*

eTable 5. Relative Excess Risk in Interaction Between New-Onset Atrial Fibrillation and Dexmedetomidine in Association With In-Hospital Mortality in Primary Cohort

Dexmedetomidine	New-onset atrial fibrillation	OR (95% CI)	RERI (95% CI)[a]
Yes	No	1 (Reference)	0.89 (0.26-1.53)
	Yes	1.04 (0.66-1.65)	
No	No	2.01 (1.63-2.50)	
	Yes	2.96 (2.32-3.77)	

CI, confidence interval; OR, odds ratio; RERI, relative excess risk due to interaction

[a]RERI = OR No dexmedetomidine&NOAF – OR No dexmedetomidine&no NOAF – OR Dexmedetomidine&NOAF +1

© 2023 Song MJ et al. *JAMA Network Open.*

eTable 6. Baseline Characteristics of Validation Cohort Before and After Propensity Score Matching

Characteristics		Before propensity score matching			After propensity score matching		
		Mean (SD)		SMD	Mean (SD)		SMD
		Dexmedetomidine (n=602)	No dexmedetomidine (n=3,131)		Dexmedetomidine (n=580)	No Dexmedetomidine (n=1,005)	
Age, y		63.99 ± 15.67	64.36 ± 16.72	0.023	64.12 ± 15.66	64.79 ± 16.63	0.041
Sex, No. (%)							
	Males	389 (64.62)	1,778 (56.79)	0.161	371 (63.97)	646 (64.28)	0.007
	Females	213 (35.38)	1353 (43.21)		209 (36.03)	359 (35.72)	
Body mass index		32.27 ± 195.09	24.35 ± 61.70	0.055	24.40 ± 20.62	27.44 ± 108.66	0.039
ICU unit, No (%)				0.847			0.162
	MICU	227 (37.71)	619 (19.77)		221 (38.10)	388 (38.61)	
	NCU	50 (8.31)	876 (27.98)		50 (8.62)	99 (9.85)	
	SICU	163 (27.08)	1,382 (44.14)		162 (27.93)	326 (32.44)	
	Other	162 (26.91)	254 (8.11)		147 (25.34)	192 (19.10)	
CCI		3.25 ± 3.28	3.60 ± 3.43	0.105	3.29 ± 3.28	3.45 ± 3.30	0.049
APACHE II		30.06 ± 8.37	23.61 ± 9.28	0.73	29.72 ± 8.25	28.70 ± 9.08	0.117
SAPS II		56.34 ± 15.46	47.21 ± 17.00	0.562	55.73 ± 15.24	54.70 ± 17.42	0.063
Vasoactive inotropic score[a]		30.45 ± 51.88	13.49 ± 32.06	0.393	29.17 ± 50.22	25.99 ± 45.47	0.066
Mechanical ventilation, No (%)		359 (59.63)	1,133 (36.19)	0.483	339 (58.45)	558 (55.52)	0.059
CKRT, No (%)		119 (19.77)	260 (8.30)	0.335	111 (19.14)	163 (16.22)	0.077
Cardiac surgery, No (%)		67 (11.13)	65 (2.08)	0.371	59 (10.17)	52 (5.17)	0.189
Mean arterial pressure, mmHg		89.66 ± 21.31	93.73 ± 21.22	0.191	89.83 ± 21.03	91.67 ± 23.86	0.082
Pulse rate, beats/min		21.16 ± 7.75	19.98 ± 6.73	0.163	21.24 ± 7.77	20.85 ± 7.14	0.051
Respiratory rate, breaths/min		100.36 ± 24.46	96.26 ± 23.01	0.173	100.48 ± 23.96	101.28 ± 24.46	0.033
Temperature, °C		36.41 ± 1.27	36.54 ± 1.10	0.106	36.43 ± 1.26	36.46 ± 1.29	0.022
Spo_2, %		95.50 ± 7.63	96.71 ± 4.83	0.190	95.76 ± 6.35	95.86 ± 6.22	0.016

APACHE, acute physiology and chronic health evaluation; SMD, standardized mean differences; ICU, intensive care unit; CCI, Charlson comorbidity index; CCU, Coronary care unit; CKRT, Continuous kidney replacement therapy; MICU, Medical intensive care unit; NCU, neurocritical care unit; SICU, Surgical intensive care unit; SOFA, Sequential Organ Failure Assessment; Spo2, oxygen saturation as measured by pulse oximetry.

[a]The vasoactive-inotropic score was calculated as follows: dopamine dose (in micrograms per kilogram per minute) + dobutamine dose (in micrograms per kilogram per minute) + 100 × epinephrine dose (in micrograms per kilogram per minute) + 10 × milrinone dose (in micrograms per kilogram per minute) + 10 000 × vasopressin dose (in international units per kilogram per minute) + 100 × norepinephrine dose (in micrograms per kilogram per minute).

© 2023 Song MJ et al. *JAMA Network Open.*

eTable 7 Outcomes of Interest in Validation Cohort

Outcomes	No. (%)			*P* value	HR (95% CI)
	**Total (n=1,585)**	**Dexmedetomidine (n=580)**	**No dexmedetomidine (n=1,005)**		
New-onset atrial atrial fibrillation	362 (22.8)	85 (14.7)	277 (27.6)	<.001	0.64 (0.49-0.83)[a]
Time to development of atrial fibrillation, median (IQR), d	0.6 (0.0-1.8)	2.1 (1.6-3.1)	0.4 (0.0-1.6)	<.001	NA
ICU length of stay, median (IQR), d	4.7 (2.9-7.9)	4.8 (3.0-8.1)	4.5 (2.9-7.9)	.490	NA
In-hospital mortality	349 (22.0)	117 (20.2)	232 (23.1)	.180	1.05 (0.84-1.32)[b]

NA, not applicable; HR, hazard ratio; ICU, intensive care unit

[a] Hazard ratio (Dexmedetomidine vs No dexmedetomidine) was estimated using a Flexible parametric survival model.

[b] Hazard ratio (Dexmedetomidine vs No dexmedetomidine) was estimated using a univariable Cox proportional hazard model.

eTable 8. Cox Proportional Hazard Model in Entire Cohort From Primary Database Before Imputation for Missing Data

**Variables**	**NOAF**					**In-hospital mortality**			
	**Univariable**		**Multivariable**			**Univariable**		**Multivariable**	
	**HR (95% CI)**	***P* value**	**HR (95% CI)**	***P* value**		**HR (95% CI)**	***P* value**	**HR (95% CI)**	***P* value**
Dexmedetomidine exposure	0.65 (0.58-0.72)	<.001	0.73 (0.65-0.81)	<.001		0.43 (0.36-0.51)	<.001	0.44 (0.37-0.53)	<.001
Age	1.05 (1.05-1.06)	<.001	1.06 (1.05-1.06)	<.001		1.02 (1.02-1.02)	<.001	1.01 (1.01-1.01)	<.001
Sex						0.93 (0.86-1.00)	0.044	0.95 (0.88-1.03)	.231
Males	1 [Reference]	NA	1 [Reference]	NA		1 [Reference]	NA	1 [Reference]	NA
Females	1.10 (1.04-1.16)	.001	1.16 (1.09-1.23)	<.001		0.93 (0.86-1.00)	0.044	0.95 (0.88-1.03)	.231
Body mass index	1.01 (1.00-1.01)	<.001	1.02 (1.02-1.02)	<.001		0.99 (0.98-0.99)	<.001	0.99 (0.98-0.99)	<.001
Race and Ethnicity									
African American	1 [Reference]	NA	1 [Reference]	NA		1 [Reference]	NA	1 [Reference]	NA
Asian	1.52 (1.24-1.86)	<.001	1.34 (1.09-1.65)	.005		1.11 (0.85-1.43)	0.448	1.02 (0.79-1.33)	.862
Hispanic	0.89 (0.71-1.12)	.336	1.03 (0.82-1.30)	.789		0.84 (0.64-1.11)	0.216	1.01 (0.77-1.34)	.93
White	1.86 (1.65-2.10)	<.001	1.42 (1.26-1.61)	<.001		1.06 (0.92-1.22)	0.449	1.14 (0.98-1.32)	.081
Other	1.46 (1.27-1.66)	<.001	1.25 (1.09-1.43)	.001		1.74 (1.50-2.03)	<.001	1.82 (1.56-2.12)	<.001
Cardiac surgery	2.07 (1.87-2.29)	<.001	1.56 (1.40-1.73)	<.001		0.94 (0.79-1.12)	0.517	0.83 (0.69-0.99)	.035
ICU Unit									
CCU	1 [Reference]	NA	1 [Reference]	NA		1 [Reference]	NA	1 [Reference]	NA
CVICU	1.17 (1.08-1.28)	<.001	1.70 (1.54-1.88)	<.001		0.25 (0.21-0.31)	<.001	0.28 (0.23-0.35)	<.001
MICU	0.65 (0.59-0.71)	<.001	0.85 (0.77-0.94)	.002		1.05 (0.92-1.19)	0.473	1.09 (0.95-1.24)	.228
SICU	0.57 (0.52-0.62)	<.001	0.87 (0.79-0.94)	.001		0.82 (0.73-0.92)	0.001	1.06 (0.94-1.20)	.346
Other	0.36 (0.30-0.44)	<.001	0.70 (0.58-0.86)	<.001		0.24 (0.17-0.35)	<.001	0.41 (0.28-0.61)	<.001
Charlson Comorbidity Index	1.15 (1.14-1.16)	<.001	1.02 (1.01-1.04)	<.001		1.14 (1.12-1.15)	<.001	1.08 (1.06-1.09)	<.001
Vasoactive Inotropic Score	1.00 (1.00-1.00)	<.001	1.00 (1.00-1.00)	.864		1.01 (1.01-1.01)	<.001	1.00 (1.00-1.00)	<.001
Mechanical ventilation	1.89 (1.74-2.06)	<.001	1.21 (1.11-1.33)	<.001		1.70 (1.51-1.92)	<.001	1.20 (1.06-1.37)	.005
CKRT	1.36 (1.10-1.68)	0.004	1.34 (1.07-1.68)	.012		2.03 (1.65-2.49)	<.001	0.71 (0.57-0.89)	.003
Sepsis	1.30 (1.23-1.38)	<.001	1.08 (1.01-1.15)	.026		2.03 (1.85-2.23)	<.001	1.17 (1.05-1.30)	.003
SOFA score	1.05 (1.05-1.06)	<.001	1.02 (1.00-1.03)	.015		1.15 (1.14-1.16)	<.001	1.13 (1.11-1.15)	<.001
SAPS II	1.03 (1.03-1.03)	<.001	1.00 (1.00-1.00)	.439		1.04 (1.04-1.05)	<.001	1.01 (1.01-1.02)	<.001

© 2023 Song MJ et al. *JAMA Network Open.*

Variables	NOAF				In-hospital mortality			
	Univariable		Multivariable		Univariable		Multivariable	
	HR (95% CI)	*P* value	HR (95% CI)	*P* value	HR (95% CI)	*P* value	HR (95% CI)	*P* value
Initial vital signs at ICU admission								
Mean arterial pressure	0.98 (0.98-0.99)	<.001	1.00 (1.00-1.00)	.176	0.98 (0.98-0.99)	<.001	1.00 (1.00-1.00)	.976
Pulse rate	1.01 (1.01-1.02)	<.001	1.02 (1.02-1.03)	<.001	1.01 (1.01-1.01)	<.001	1.00 (1.00-1.01)	<.001
Respiratory rate	1.02 (1.01-1.02)	<.001	1.00 (0.99-1.00)	.145	1.02 (1.02-1.03)	<.001	1.01 (1.00-1.01)	.005
Temperature	0.82 (0.79-0.85)	<.001	0.88 (0.85-0.92)	<.001	0.83 (0.79-0.87)	<.001	0.87 (0.83-0.91)	<.001
Spo$_2$	0.98 (0.97-0.98)	<.001	0.99 (0.99-0.99)	<.001	0.98 (0.97-0.98)	<.001	1.00 (0.99-1.00)	.176
Laboratory data on day of ICU admission								
pH	0.54 (0.42-0.70)	<.001	0.60 (0.36-1.02)	.058	0.09 (0.07-0.12)	<.001	0.60 (0.33-1.08)	.089
po$_2$	1.00 (1.00-1.00)	<.001	1.00 (1.00-1.00)	.714	1.00 (1.00-1.00)	<.001	1.00 (1.00-1.00)	<.001
pCo$_2$	1.01 (1.00-1.01)	<.001	0.99 (0.99-1.00)	.001	1.01 (1.00-1.01)	<.001	1.00 (1.00-1.00)	.932
White blood cell	1.00 (1.00-1.01)	<.001	1.00 (1.00-1.00)	.431	1.00 (1.00-1.01)	<.001	1.00 (1.00-1.00)	.297
Hemoglobin	0.93 (0.92-0.95)	<.001	1.02 (1.01-1.04)	.002	0.96 (0.95-0.98)	<.001	1.03 (1.01-1.05)	.001
Platelet	1.00 (1.00-1.00)	<.001	1.00 (1.00-1.00)	<.001	1.00 (1.00-1.00)	<.001	1.00 (1.00-1.00)	.002
Creatinine	1.04 (1.03-1.06)	<.001	0.97 (0.94-1.00)	.025	1.06 (1.04-1.07)	<.001	0.88 (0.85-0.91)	<.001
Blood urea nitrogen	1.01 (1.01-1.01)	<.001	1.01 (1.00-1.01)	<.001	1.01 (1.01-1.01)	<.001	1.00 (1.00-1.00)	.769
Lactate	1.02 (1.02-1.03)	<.001	1.01 (1.00-1.02)	.241	1.05 (1.05-1.05)	<.001	1.03 (1.02-1.04)	<.001
Sodium	0.99 (0.98-0.99)	<.001	1.00 (0.99-1.01)	.739	1.02 (1.01-1.03)	<.001	1.03 (1.01-1.04)	<.001
Chloride	0.99 (0.99-1.00)	<.001	0.98 (0.97-0.99)	<.001	1.00 (0.99-1.00)	.587	0.98 (0.97-0.99)	<.001
Potassium	1.12 (1.09-1.15)	<.001	1.02 (0.99-1.06)	.24	1.17 (1.13-1.21)	<.001	1.02 (0.97-1.06)	.508
Calcium	1.00 (0.97-1.03)	.942	0.96 (0.92-0.99)	.023	0.90 (0.86-0.94)	<.001	1.03 (0.98-1.08)	.223
Bicarbonate	1.01 (1.00-1.01)	.067	1.02 (1.01-1.03)	<.001	0.95 (0.94-0.96)	<.001	0.98 (0.97-1.00)	.023
Magnesium	1.80 (1.67-1.94)	<.001	1.30 (1.19-1.42)	<.001	1.15 (1.04-1.28)	.009	1.45 (1.28-1.64)	<.001

CCU, Coronary care unit; CKRT, Continuous kidney replacement therapy; CVICU, Cardiovascular intensive care unit; ICU, intensive care unit; MICU, Medical intensive care unit; NOAF; new-onset atrial fibrillation; pCo2, partial pressure of carbon dioxide; po2, partial pressure of oxygen; SAPS, Simplified Acute Physiology Score; SICU, Surgical intensive care unit; SOFA, Sequential Organ Failure Assessment; Spo$_2$, oxygen saturation as measured by pulse oximetry.

© 2023 Song MJ et al. *JAMA Network Open.*

eTable 9. Cox Proportional Hazard Model in Entire Cohort From Primary Database After Imputation for Missing Data

Variables		NOAF					In-hospital mortality			
		**Univariable**		**Multivariable**			**Univariable**		**Multivariable**	
		**HR (95% CI)**	***P* value**	**HR (95% CI)**	***P* value**		**HR (95% CI)**	***P* value**	**HR (95% CI)**	***P* value**
Dexmedetomidine exposure		0.65 (0.58-0.72)	<.001	0.73 (0.65-0.81)	<.001		0.43 (0.36-0.51)	<.001	0.44 (0.37-0.53)	<.001
Age		1.05 (1.05-1.06)	<.001	1.06 (1.05-1.06)	<.001		1.02 (1.02-1.02)	<.001	1.01 (1.01-1.01)	<.001
Sex										
	Males	1 [Reference]	NA	1 [Reference]	NA		1 [Reference]	NA	1 [Reference]	NA
	Females	1.10 (1.04-1.16)	.001	1.16 (1.09-1.23)	<.001		0.93 (0.86-1.00)	.044	0.95 (0.88-1.03)	.231
Body mass index		1.01 (1.00-1.01)	<.001	1.02 (1.02-1.02)	<.001		0.99 (0.98-0.99)	<.001	0.99 (0.98-0.99)	<.001
Race and Ethnicity										
	African American									
	Asian	1.52 (1.24-1.86)	<.001	1.34 (1.09-1.65)	.005		1.11 (0.85-1.43)	.448	1.02 (0.79-1.33)	.862
	Hispanic	0.89 (0.71-1.12)	.336	1.03 (0.82-1.30)	.789		0.84 (0.64-1.11)	.216	1.01 (0.77-1.34)	.93
	White	1.86 (1.65-2.10)	<.001	1.42 (1.26-1.61)	<.001		1.06 (0.92-1.22)	.449	1.14 (0.98-1.32)	.081
	Other	1.46 (1.27-1.66)	<.001	1.25 (1.09-1.43)	.001		1.74 (1.50-2.03)	<.001	1.82 (1.56-2.12)	<.001
Cardiac surgery		2.07 (1.87-2.29)	<.001	1.56 (1.40-1.73)	<.001		0.94 (0.79-1.12)	.517	0.83 (0.69-0.99)	.035
ICU Unit										
	CCU									
	CVICU	1.17 (1.08-1.28)	<.001	1.70 (1.54-1.88)	<.001		0.25 (0.21-0.31)	<.001	0.28 (0.23-0.35)	<.001
	MICU	0.65 (0.59-0.71)	<.001	0.85 (0.77-0.94)	.002		1.05 (0.92-1.19)	.473	1.09 (0.95-1.24)	.228
	SICU	0.57 (0.52-0.62)	<.001	0.87 (0.79-0.94)	.001		0.82 (0.73-0.92)	.001	1.06 (0.94-1.20)	.346
	Other	0.36 (0.30-0.44)	<.001	0.70 (0.58-0.86)	<.001		0.24 (0.17-0.35)	<.001	0.41 (0.28-0.61)	<.001
Charlson Comorbidity Index		1.15 (1.14-1.16)	<.001	1.02 (1.01-1.04)	<.001		1.14 (1.12-1.15)	<.001	1.08 (1.06-1.09)	<.001
Vasoactive Inotropic Score		1.00 (1.00-1.00)	<.001	1.00 (1.00-1.00)	.864		1.01 (1.01-1.01)	<.001	1.00 (1.00-1.00)	<.001
Mechanical ventilation		1.89 (1.74-2.06)	<.001	1.21 (1.11-1.33)	<.001		1.70 (1.51-1.92)	<.001	1.20 (1.06-1.37)	.005
CKRT		1.36 (1.10-1.68)	0.004	1.34 (1.07-1.68)	.012		2.03 (1.65-2.49)	<.001	0.71 (0.57-0.89)	.003
Sepsis		1.30 (1.23-1.38)	<.001	1.08 (1.01-1.15)	.026		2.03 (1.85-2.23)	<.001	1.17 (1.05-1.30)	.003
SOFA score		1.05 (1.05-1.06)	<.001	1.02 (1.00-1.03)	.015		1.15 (1.14-1.16)	<.001	1.13 (1.11-1.15)	<.001
SAPS II		1.03 (1.03-1.03)	<.001	1.00 (1.00-1.00)	.439		1.04 (1.04-1.05)	<.001	1.01 (1.01-1.02)	<.001

© 2023 Song MJ et al. *JAMA Network Open.*

Variables	NOAF					In-hospital mortality			
	Univariable		Multivariable			Univariable		Multivariable	
	HR (95% CI)	*P* value	HR (95% CI)	*P* value		HR (95% CI)	*P* value	HR (95% CI)	*P* value
Initial vital signs at ICU admission									
Mean arterial pressure	0.98 (0.98-0.99)	<.001	1.00 (1.00-1.00)	.176		0.98 (0.98-0.99)	<.001	1.00 (1.00-1.00)	.976
Pulse rate	1.01 (1.01-1.02)	<.001	1.02 (1.02-1.03)	<.001		1.01 (1.01-1.01)	<.001	1.00 (1.00-1.01)	<.001
Respiratory rate	1.02 (1.01-1.02)	<.001	1.00 (0.99-1.00)	.145		1.02 (1.02-1.03)	<.001	1.01 (1.00-1.01)	.005
Temperature	0.82 (0.79-0.85)	<.001	0.88 (0.85-0.92)	<.001		0.83 (0.79-0.87)	<.001	0.87 (0.83-0.91)	<.001
Spo_2	0.98 (0.97-0.98)	<.001	0.99 (0.99-0.99)	<.001		0.98 (0.97-0.98)	<.001	1.00 (0.99-1.00)	.176
Laboratory data on day of ICU admission									
pH	0.54 (0.42-0.70)	<.001	0.60 (0.36-1.02)	.058		0.09 (0.07-0.12)	<.001	0.60 (0.33-1.08)	.089
po_2	1.00 (1.00-1.00)	<.001	1.00 (1.00-1.00)	.714		1.00 (1.00-1.00)	<.001	1.00 (1.00-1.00)	<.001
pCo_2	1.01 (1.00-1.01)	<.001	0.99 (0.99-1.00)	.001		1.01 (1.00-1.01)	<.001	1.00 (1.00-1.00)	.932
White blood cell	1.00 (1.00-1.01)	<.001	1.00 (1.00-1.00)	.431		1.00 (1.00-1.01)	<.001	1.00 (1.00-1.00)	.297
Hemoglobin	0.93 (0.92-0.95)	<.001	1.02 (1.01-1.04)	.002		0.96 (0.95-0.98)	<.001	1.03 (1.01-1.05)	.001
Platelet	1.00 (1.00-1.00)	<.001	1.00 (1.00-1.00)	<.001		1.00 (1.00-1.00)	<.001	1.00 (1.00-1.00)	.002
Creatinine	1.04 (1.03-1.06)	<.001	0.97 (0.94-1.00)	.025		1.06 (1.04-1.07)	<.001	0.88 (0.85-0.91)	<.001
Blood urea nitrogen	1.01 (1.01-1.01)	<.001	1.01 (1.00-1.01)	<.001		1.01 (1.01-1.01)	<.001	1.00 (1.00-1.00)	.769
Lactate	1.02 (1.02-1.03)	<.001	1.01 (1.00-1.02)	.241		1.05 (1.05-1.05)	<.001	1.03 (1.02-1.04)	<.001
Sodium	0.99 (0.98-0.99)	<.001	1.00 (0.99-1.01)	.739		1.02 (1.01-1.03)	<.001	1.03 (1.01-1.04)	<.001
Chloride	0.99 (0.99-1.00)	<.001	0.98 (0.97-0.99)	<.001		1.00 (0.99-1.00)	.587	0.98 (0.97-0.99)	<.001
Potassium	1.12 (1.09-1.15)	<.001	1.02 (0.99-1.06)	.240		1.17 (1.13-1.21)	<.001	1.02 (0.97-1.06)	.508
Calcium	1.00 (0.97-1.03)	.942	0.96 (0.92-0.99)	.023		0.90 (0.86-0.94)	<.001	1.03 (0.98-1.08)	.223
Bicarbonate	1.01 (1.00-1.01)	.067	1.02 (1.01-1.03)	<.001		0.95 (0.94-0.96)	<.001	0.98 (0.97-1.00)	.023
Magnesium	1.80 (1.67-1.94)	<.001	1.30 (1.19-1.42)	<.001		1.15 (1.04-1.28)	.009	1.45 (1.28-1.64)	<.001

CCU, Coronary care unit; CKRT, Continuous kidney replacement therapy; CVICU, Cardiovascular intensive care unit; ICU, intensive care unit; MICU, Medical intensive care unit; NOAF; new-onset atrial fibrillation; pCo_2, partial pressure of carbon dioxide; po_2, partial pressure of oxygen; SAPS, Simplified Acute Physiology Score; SICU, Surgical intensive care unit; SOFA, Sequential Organ Failure Assessment; Spo_2, oxygen saturation as measured by pulse oximetry.

eTable 10. Association Between Dexmedetomidine Infusion and Outcomes in Different Models.

**Models**		**Hazard Ratio**	**Lower 95% CI**	**Upper 95% CI**	***P* value**
In primary cohort [a]					
New-onset atrial fibrillation					
	Multivariable Cox regression	0.7	0.62	0.81	<.001
	Multivariable Cox regression after imputation	0.72	0.65	0.8	<.001
	IPTW	0.52	0.45	0.6	<.001
In-hospital mortality					
	Multivariable Cox regression	0.48	0.39	0.59	<.001
	Multivariable Cox regression after imputation	0.48	0.4	0.57	<.001
	IPTW	0.54	0.43	0.67	<.001
In validation cohort [b]					
New-onset atrial fibrillation					
	Multivariable Cox regression	0.48	0.37	0.63	<.001
	Multivariable Cox regression after imputation	0.43	0.34	0.55	<.001
	IPTW	0.52	0.41	0.65	<.001
In-hospital mortality					
	Multivariable Cox regression	1.05	0.82	1.34	.712
	Multivariable Cox regression after imputation	0.98	0.79	1.21	.850
	IPTW	1.17	0.95	1.44	.130

CI, confidence interval; IPTW, inverse probability of treatment weighting
[a] Covariates used in the analyses are shown in eTable 2.
[b] Covariates used in the analyses are shown in eTable 3.

© 2023 Song MJ et al. *JAMA Network Open.*

eTable 11. Sensitivity Analyses for Patients by Duration of Dexmedetomidine Administration in Primary Cohort

Duration of dexmedetomidine administration	Number of patients after propensity score matching [a]		Outcome	HR (95% CI)	*P* value
	Dexmedetomidine	No dexmedetomidine			
< 48 hr	1,596	1,596	NOAF	0.79 (0.67-0.92)	.002
			In-hospital mortality	0.40 (0.30-0.52)	<.001
≥ 48 hr, < 96 hr	242	242	NOAF	0.61 (0.41-0.92)	.019
			In-hospital mortality	0.51 (0.30-0.88)	.016
≥ 96 hr	256	256	NOAF	0.60 (0.43-0.93)	.022
			In-hospital mortality	0.47 (0.27-0.72)	.001

CI, confidence interval; HR, hazard ratio; NOAF, new-onset atrial fibrillation

[a]1:1 propensity score matching adjusting for covariates as per eTable 2.

© 2023 Song MJ et al. *JAMA Network Open.*

eTable 12. Sensitivity Analyses for Patients by Calendar Period of Intensive Care Unit Admission

Year of admission	Number of patients after propensity score matching		Outcome	HR (95% CI)	*P* value
	**Dexmedetomidine**	**No dexmedetomidine**			
In primary cohort [a]					
2008–2013	579	1,128	NOAF	0.74 (0.60-0.91)	<.001
			In-hospital mortality	0.41 (0.25-0.67)	<.001
2014–2016	490	951	NOAF	0.74 (0.57-0.94)	.010
			In-hospital mortality	0.37 (0.23-0.58)	<.001
2017–2019	531	1,013	NOAF	0.59 (0.46-0.76)	<.001
			In- hospital mortality	0.45 (0.31-0.64)	<.001
In validation cohort [b]					
2013.04–2015.06	236	441	NOAF	0.48 (0.33-0.70)	<.001
			In- hospital mortality	0.94 (0.68-1.31)	.710
2015.07–2017.12	325	507	NOAF	0.46 (0.32-0.65)	<.001
			In- hospital mortality	1.15 (0.83-1.58)	.400

CI, confidence interval; HR, hazard ratio; NOAF, new onset atrial fibrillation

[a] 1:2 propensity score matching adjusting for covariates as per eTable 2.

[b] 1:2 propensity score matching adjusting for covariates as per eTable 3.

© 2023 Song MJ et al. *JAMA Network Open.*

eFigure 1. Absolute Standardized Mean Differences of Individual Covariates Before and After Propensity Score Matching in Primary Cohort

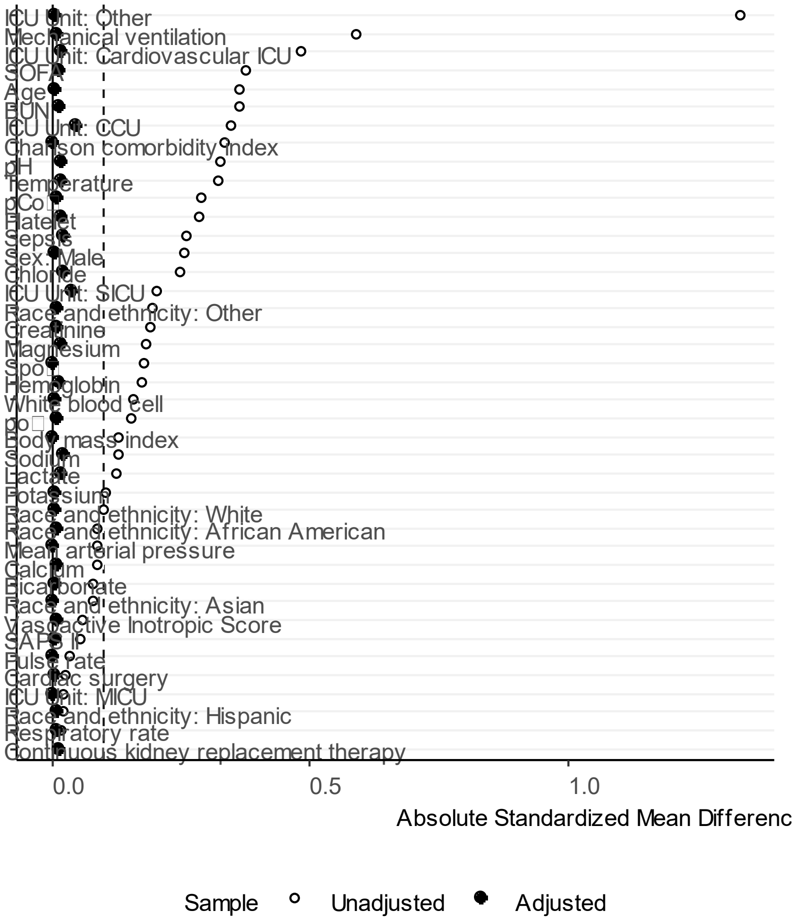

BUN; blood urea nitrogen; CCU; coronary care unit; ICU, intensive care unit; MICU, Medical intensive care unit; pCo2, partial pressure of carbon dioxide; po2, partial pressure of oxygen; SAPS, Simplified Acute Physiology Score; SICU, Surgical intensive care unit; SOFA, Sequential Organ Failure Assessment; Spo_2, oxygen saturation as measured by pulse oximetry.

© 2023 Song MJ et al. *JAMA Network Open.*

eFigure 2. Dose-Response Association Between Quartiles of Dexmedetomidine Dose and Incidence of New-Onset Atrial Fibrillation

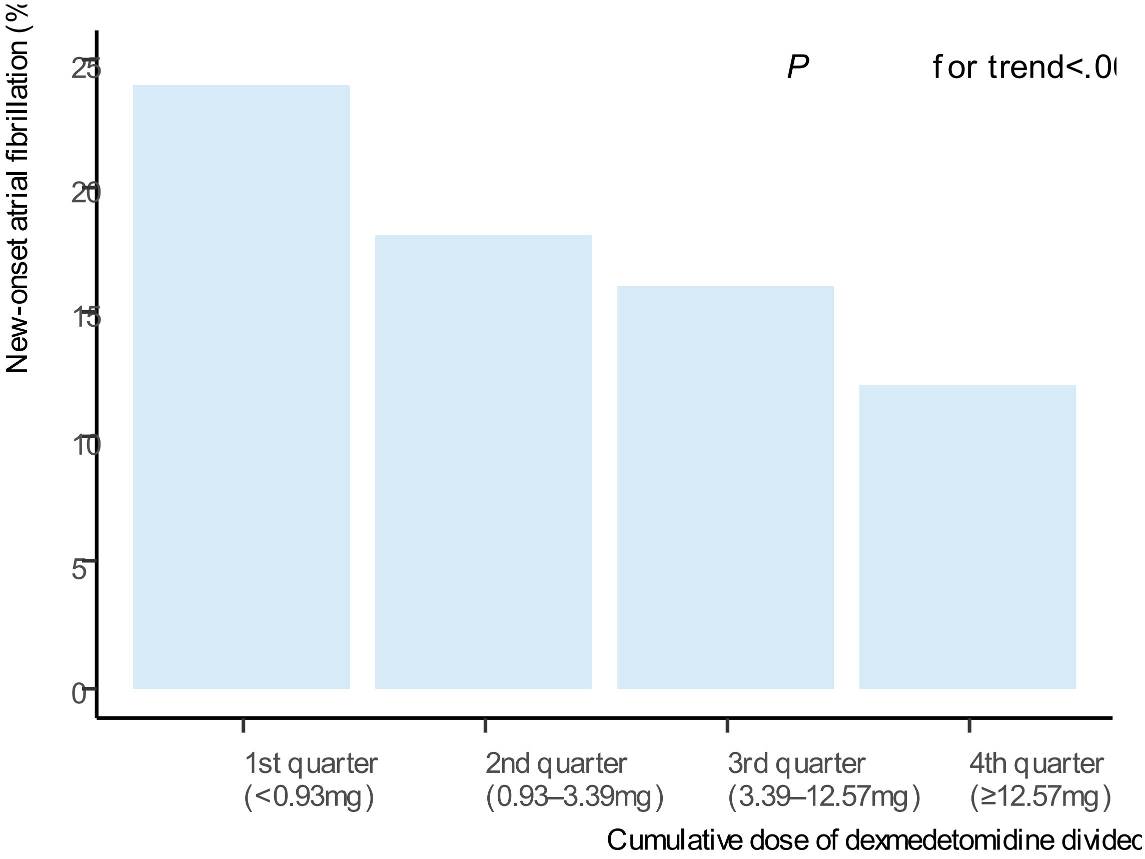

© 2023 Song MJ et al. *JAMA Network Open.*

eFigure 3. Study Flowchart for Validation Cohort

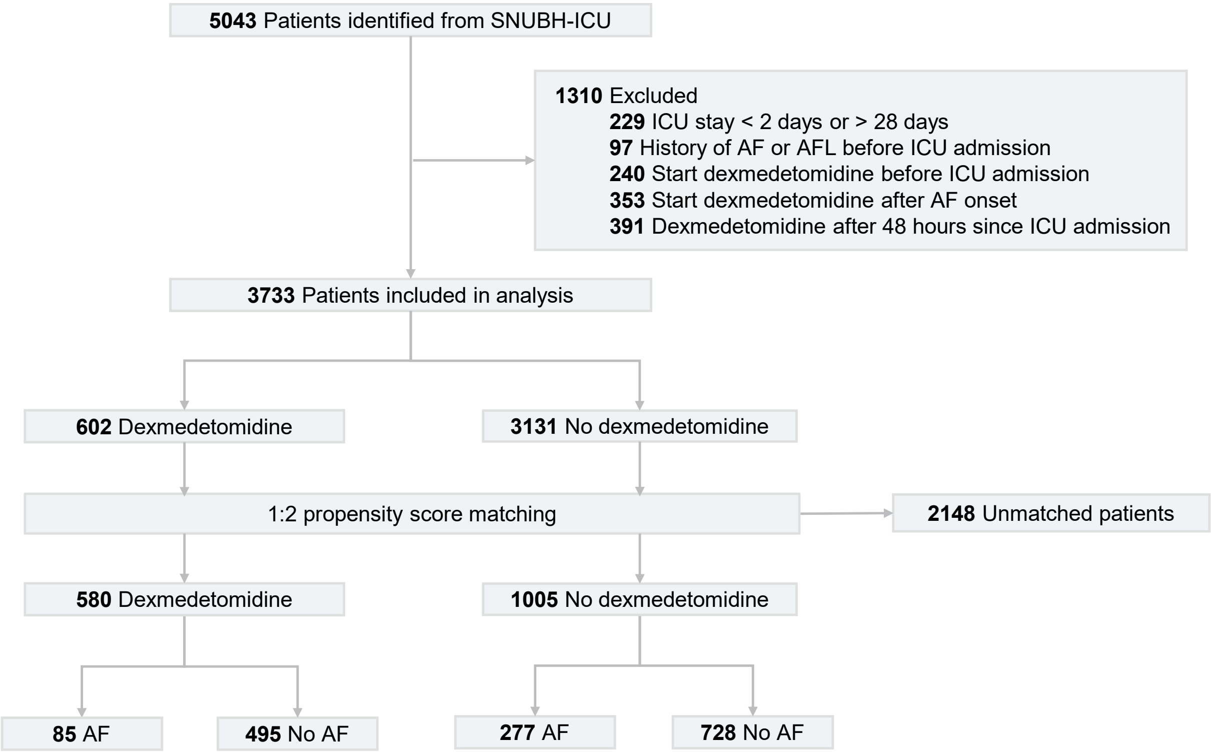

AF, atrial fibrillation; AFL, atrial flutter; ICU, intensive care unit; NOAF, new-onset atrial fibrillation

© 2023 Song MJ et al. *JAMA Network Open.*

eFigure 4. Absolute Standardized Mean Differences of Individual Covariates Before and After Propensity Score Matching in Validation Cohort

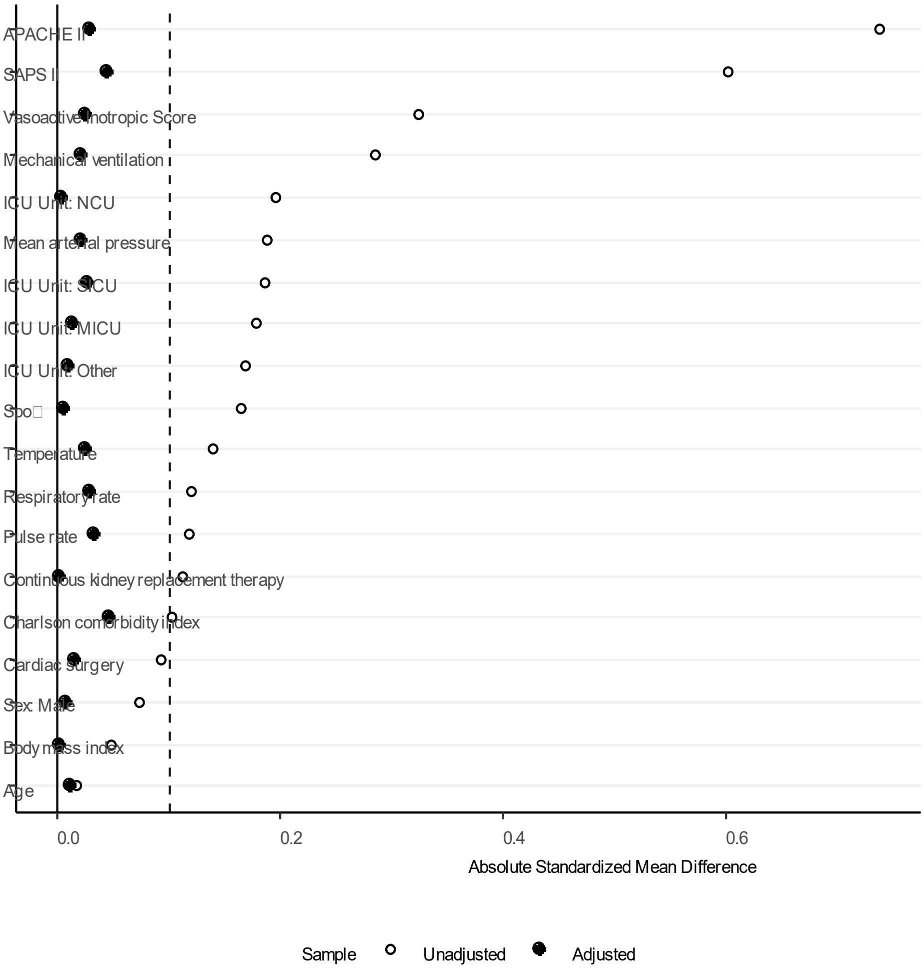

APACHE, acute physiology and chronic health evaluation; ICU, intensive care unit; MICU, Medical intensive care unit; NCU, neurocritical care unit; SAPS, Simplified Acute Physiology Score; SICU, Surgical intensive care unit; Spo2, oxygen saturation as measured by pulse oximetry.

© 2023 Song MJ et al. *JAMA Network Open.*

eFigure 5. Incidence of New-Onset Atrial Fibrillation and Kaplan-Meier Survival Plot for Dexmedetomidine Use in Validation Cohort

A. Cumulative incidence of new-onset atrial fibrillation for dexmedetomidine use

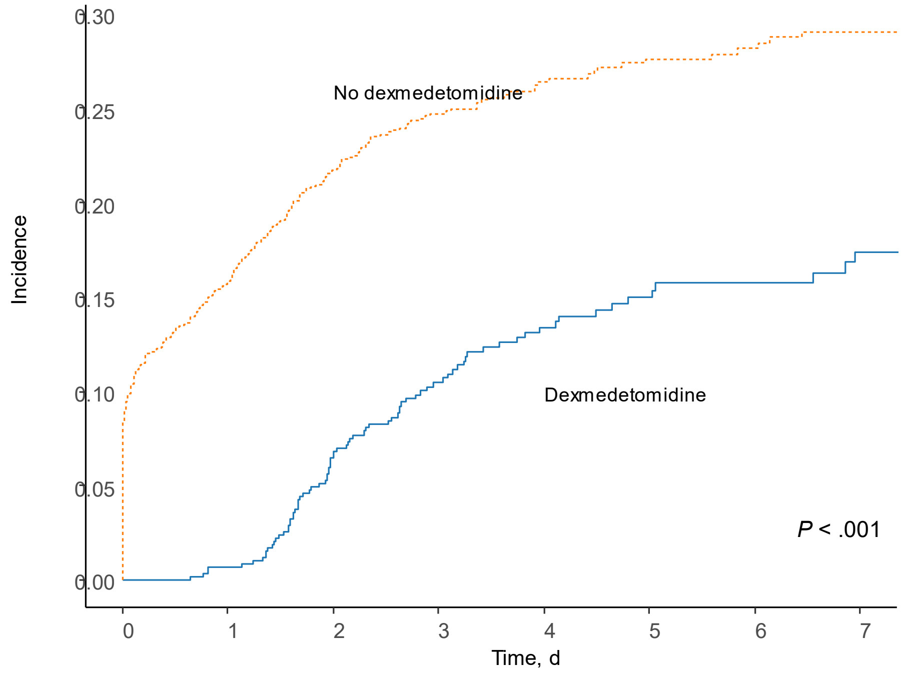

idine	594	590	555	411	304	233	190	154
omidine	893	757	703	490	373	287	225	180

© 2023 Song MJ et al. *JAMA Network Open.*

B. Kaplan-Meier survival plot for dexmedetomidine use

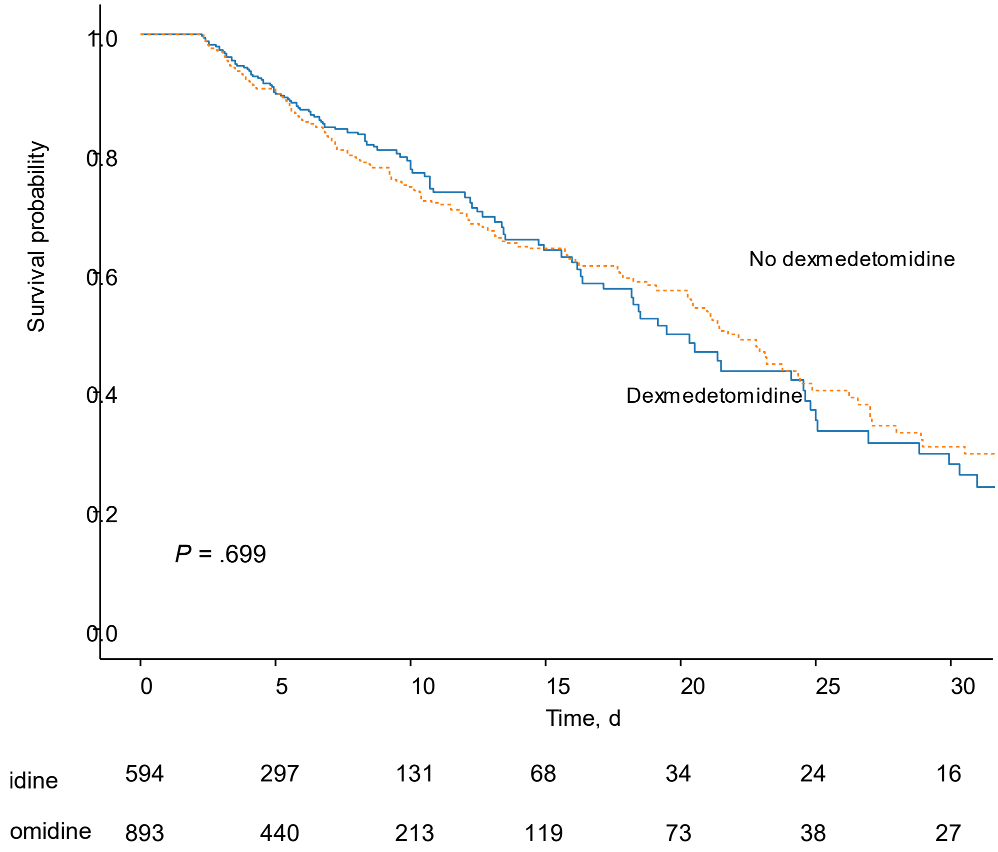

© 2023 Song MJ et al. *JAMA Network Open.*

eReferences

1. Koponen T, Karttunen J, Musialowicz T, Pietiläinen L, Uusaro A, Lahtinen P. Vasoactive-inotropic score and the prediction of morbidity and mortality after cardiac surgery. *Br J Anaesth*. 2019;122(4):428-436, https://doi.org/10.1016/j.bja.2018.12.019.

2. Singer M, Deutschman CS, Seymour CW, et al. The Third International Consensus Definitions for Sepsis and Septic Shock (Sepsis-3). *JAMA*. 2016;315(8):801-810, https://doi.org/10.1001/jama.2016.0287.

3. KJ R. Measuring interactions; in Epidemiology: An Introduction. *Oxford University Press;* 2002:168–180.

4. Andersson T, Alfredsson L, Källberg H, Zdravkovic S, Ahlbom A. Calculating measures of biological interaction. *Eur J Epidemiol*. 2005;20(7):575-579, https://doi.org/10.1007/s10654-005-7835-x.

5. Knol MJ, VanderWeele TJ, Groenwold RH, Klungel OH, Rovers MM, Grobbee DE. Estimating measures of interaction on an additive scale for preventive exposures*. Eur J Epidemiol*. 2011;26(6):433-438, https://doi.org/10.1007/s10654-011-9554-9.

© 2023 Song MJ et al. *JAMA Network Open.*
