# Supplementary material for: Association of Dexmedetomidine With New-Onset Atrial Fibrillation in Patients With Critical Illness
Source: JAMA Netw Open. 2023 Apr 25;6(4):e239955. doi: 10.1001/jamanetworkopen.2023.9955 (PMC10130948; doi:10.1001/jamanetworkopen.2023.9955)
Supplement: Supplement 2. — Data Sharing Statement [file jamanetwopen-e239955-s002.pdf]

Data Sharing Statement

Song. Association of Dexmedetomidine With New-Onset Atrial Fibrillation in Patients With Critical Illness. *JAMA Netw Open*. Published online April 25, 2023. doi:10.1001/jamanetworkopen.2023.9955

Data
Data available: Yes
Data types: Deidentified participant data
How to access data: MIMIC-IV data are publicly available. SNUBH-ICU data are available upon request to the corresponding author.
When available: With publication

Supporting Documents
Document types: Statistical/analytic code
How to access documents: Statistical code is available on reasonable request to the corresponding author.
When available: With publication

Additional Information
Who can access the data: Anyone requesting the data.
Types of analyses: For any purpose.
Mechanisms of data availability: The data will be available with investigator support.

© 2023 Song MJ et al. *JAMA Network Open*.
